# Evidence for widespread cytoplasmic structuring into mesoscale condensates

**DOI:** 10.1038/s41556-024-01363-5

**Published:** 2024-02-29

**Authors:** Felix C. Keber, Thao Nguyen, Andrea Mariossi, Clifford P. Brangwynne, Martin Wühr

**Affiliations:** 1 Department of Molecular Biology, Princeton University, Princeton, NJ, USA; 2 Department of Chemical and Biological Engineering, Princeton University, Princeton, NJ, USA; 3 Lewis-Sigler Institute for Integrative Genomics, Princeton University, Princeton, NJ, USA; 4 Howard Hughes Medical Institute, Princeton University, Princeton, NJ, USA; 5 Omenn-Darling Bioengineering Institute, Princeton University, Princeton, NJ, USA

## Abstract

Compartmentalization is an essential feature of eukaryotic life and is achieved both via membrane-bound organelles, such as mitochondria, and membrane-less biomolecular condensates (BMCs), such as the nucleolus. Known BMCs typically exhibit liquid-like properties and are visualized by microscopy, on the scale of ~1 μm^1,2^. They have been studied mostly by microscopy, examining select individual proteins. To date, several dozen BMCs are known, serving a multitude of functions, e.g. in the regulation of transcription^3^, RNA processing^4^, or signaling^5,6^, and their malfunction can cause diseases^7,8^. However, it remains unclear to what extent BMCs are utilized in cellular organization and at what length-scale they typically form. Here, we examine native cytoplasm from Xenopus egg extract on a global scale with quantitative proteomics, filtration, size exclusion, and dilution experiments. These assays reveal that at least 18% of the proteome is organized into mesoscale BMCs at the scale of ~100 nm, and appear to be stabilized by RNA or gelation. We confirmed mesoscale sizes via imaging below the diffraction limit, by investigating protein permeation into porous substrates with defined pore sizes. Our results show that eukaryotic cytoplasm organizes extensively via BMCs, but at surprisingly short length scales.

Protein components of BMCs have typically been identified via imaging, co-isolation, or proximity labeling^9–14^. However, these approaches require prior knowledge of at least one constituent of the assembly. Moreover, imaging approaches favor the detection of large (~1 μm) assemblies, due to the diffraction limit of light microscopy, and are often facilitated using overexpression of labeled proteins, with potential impacts on native condensate structure. To date, these approaches have identified about ~100 “scaffold” proteins suggested to drive liquid-liquid phase separation (LLPS)^15^, which is ~0.5% of the human proteome. However, based on sequence similarity to these proteins, it has been speculated that as much as 20% of the proteome is functionally involved in LLPS^16^. We developed methods to measure what part of the proteome is organized in BMCs, and at what length-scale these typically form.

To assay the physical properties of protein assemblies throughout the native cytoplasm, we sought to combine filtration experiments of undiluted cytoplasm with quantitative proteomics. We reasoned that we could identify BMCs based on their behavior upon filtration. When encountering a pore, assemblies smaller than the pore diameter should pass freely. However, the permeation of assemblies larger than the pore will depend on their material properties. While large rigid bodies will not pass, deformable assemblies can squeeze through pores. Thus, size and viscoelastic properties will determine the passage time, establishing a chromatography-like process (Fig. 1a). At the earliest time, the filtrate should contain only solvent and freely passing proteins, since assemblies that must squeeze through pores spend more time in the porous medium, and their elution onset is later; such behavior can be described in a simplistic model (Supplementary Figures 1–3). Thus, by comparing filtrates at different times, we sought to identify assemblies that exhibit squeezing behavior. We performed microscopy experiments as proof of principle (Supplementary Figures 4 and 5).

To study the organization of near-native cytoplasm, we chose lysate from eggs of the frog *Xenopus laevis*, which provides easy access to large amounts of undiluted cytoplasm in a near-native state. Eggs are naturally arrested in metaphase; thus, the nuclear proteins are in the cytoplasm. *X. laevis* extracts have been a powerful model for studying biochemistry in the cytoplasm, that allows observing protein interactions in a close-to-native environment, including complex processes like the formation of nuclei or spindles^17,18^. Eggs are crushed in an extract preparation spin. We verified that this spin did not sediment proteins known to be involved in BMCs (Extended Data Fig. 1). Cytoplasmic extract was then centrifuged against polyethersulfone filter membranes with a defined particle size cutoff diameter ($d_{pore}$). We analyzed the filtrates of various experimental conditions by multiplexed proteomics, quantifying each protein’s concentration relative to the input^19–21^, where proteins with the earliest onset served for normalization (Fig. 1b)

We compared the permeation of all proteins at fixed pore size ($d_{pore}=30\;nm$) at exemplary stages in the elution process ($t_{1}$ and ${\;t}_{2}$). At both time points, the proteins’ concentrations relative to the input display a broad spectrum, ranging from free passage to heavy retention (Fig. 1c). The data displays a sharp top edge slightly below the identity line that originates from the systematically higher elution at $t_{2}$. We observe two major regimes: 1) A large part of the proteome shows approximately the same concentration at ${\;t}_{1}$ and ${\;t}_{2}$ (following close to identity line). This includes proteins that are organized smaller than the pores (close to the origin). In our passage-time model, these protein species’ elution has started long before ${\;t}_{1}$, thus ${\;t}_{1}$ and ${\;t}_{2}$ both sample the flat part of the curve (close to the dark blue solvent curve, Fig. 1a). 2) For many other proteins, elution is increased at ${\;t}_{2}$ (significantly below the identity line). This corresponds to sampling the steep part of the curves in our model (blue or green curves, Fig. 1a), indicating a longer passage time. We find that annotated canonical large protein complexes, which we do not expect to exhibit “squeezing” behavior, exhibit a preponderance along the top edge of the data, closer to the identity line (magenta symbols Fig. 1c); the deviation from the identity line likely reflects the influence of their size alone. By contrast, we find that proteins known to be associated with LLPS are enriched farther from the identity line (green symbols Fig. 1c; see Supplementary Table1)^22–26^, consistent with their presence within liquid-like assemblies, that slowly squeeze through pores. Executing the experiment with a larger pore size ($d_{pore}=100\;nm$) yields qualitatively similar results (Extended Data Fig. 2a). To characterize the squeezing behavior, we measure each protein’s distance from the top edge close to the identity line. This “squeezing score” displays significant differences between known LLPS proteins and the whole proteome, as reflected in the receiver operating characteristic (ROC) (Fig. 1d, and Extended Data Fig. 2b for $d_{pore}=100\;nm$). These findings suggest that filtration chromatography probes physical properties of the cytoplasm on the mesoscale and could be used to identify novel BMCs and their components.

A characteristic property of phase-separated BMCs is that they typically form via dynamic, multivalent interactions, and disassemble below a particular saturation concentration^1,2,27,28^. To test whether altering concentration impacts the apparent mesoscale cytoplasmic organization, we diluted cell extracts to various extents and examined their filtration behavior at fixed pore size ($d_{pore}=100\;nm$) (Fig. 2a). Remarkably, at a dilution of only 1.4-fold, known LLPS proteins show significantly different permeation behavior compared with canonical larger complexes or the entire proteome (Fig. 2b, c). LLPS proteins exhibit a progressive increase in permeation with dilution, which likely originates from shrinking BMC sizes as concentrations approach and cross below different saturation thresholds. Interestingly, however, even at high dilution factors, we do not observe unhindered permeation, as may be expected upon full dissolution of assemblies. Similarly, a complementary assay applying hard spins to cytoplasm detected sedimentation of LLPS proteins as expected for large assemblies; however, dilution did not abolish this sedimentation (Extended Data Fig. 3). These data suggest that while these mesoscale condensates can be partially dissolved upon dilution, they also exhibit partially solid-like character that may indicated stable cores, potentially formed by specific protein-protein interactions, gelation, or binding to RNA molecules^29,30^.

RNAs are long polymers that are usually coated in RNA-binding proteins (RBPs). Together with their protein binding partners, RNAs often help drive formation condensates, which can thereby impact associated functions, such as messenger RNA (mRNA) translation efficiency and stability. Many of the proteins that exhibit differential filtration behavior in our assay are also RBPs. Indeed, RBPs were preferentially retained and squeezable, especially if they were annotated to be involved in LLPS (Extended Data Fig. 4a, b). We thus sought to further examine the role of RNA in this emergent cytoplasmic organization. When we repeated the filtration experiment after treating the extract with RNase, the elution of RBPs was increased, with the effect strongest for LLPS-associated proteins (Extended Data Fig. 4c). These findings suggest that RNA-RBP interactions play a major role in the time-dependent squeezing behavior and are consistent with the significant presence of RNA in many or all known condensates.

To further examine which RNAs contribute to squeezability, we performed RNA-seq of filtrates at different elution times. We detect RNA exhibiting comparable behavior to what we observed on the protein level (Extended Data Fig. 5a, Fig. 1c). Remarkably, more than 15% of all RNAs showed behavior consistent with organization via BMCs. Intriguingly, these RNAs were enriched for developmental regulations, while RNAs of housekeeping genes^31^ rarely showed high retention (Extended Data Fig. 5b–d). This suggests that mRNAs that undergo transient translation like germ granules^32,33^ might be particularly prone to organization via BMCs.

Cellular BMCs to date have been reported mainly on the micrometer length scale. However, there is growing evidence for much smaller structures^34,35^. To investigate the length scale of the liquid-like organization in our system, we compared the elution behavior at different pore diameters (30/100/200 nm). At the largest pore size of 200 nm, we observe an overall higher permeation with very few proteins retained (Fig. 3a). Notable exceptions include retention of mitochondrial proteins, which is not surprising (Fig. 3a). The strikingly higher retention as the pore size is decreased to 100 nm suggests this is a characteristic length scale of liquid-like organization in the cytoplasm. This is further supported by filtration experiments in a complementary setup, using polymer mesh filters and gravity flow to avoid force-induced squeezing (Extended Data Fig. 6). These data suggest the widespread presence of liquid-like cytoplasmic assemblies on the sub-micrometer scale, involving a broad swath of the proteome.

To further validate our findings of liquid-like organization on surprisingly short length-scales, we employed an experimentally orthogonal light microscopy assay, that investigates the size of cytoplasmic assemblies below the diffraction limit. We selected proteins exhibiting different filtration behavior and expressed GFP-tagged versions by doping the cytoplasm with the corresponding mRNA. Interestingly, at our low expression levels of only a few tens of nM, we can detect fluorescence, but none of the investigated proteins - including established LLPS proteins like HNRNPA1^36^ or CIRBP^37^ - show any signs of assemblies on the micron-scale (Extended Data Fig. 7a). However, diffusion constant measurements by fluorescence correlation spectroscopy suggested organization into small assemblies (Extended Data Fig. 7b). To interrogate potential assemblies on smaller length scales, we equilibrated the lysates with size exclusion beads (Fig. 3b). The size exclusion of biomolecular assemblies by a bead’s polymer matrix reduces the concentration inside the bead $c_{in}$ compared to the outside $c_{out}$. The concentration ratio $c_{in}\;/c_{out}\;\;$ at different cutoff sizes (~7.7/15/29/53 nm) serves as a proxy for the cumulative assembly size distribution. We measure $c_{in}$ and $c_{out}$ by their GFP intensity and correct $c_{in}$ for the excluded volume in the beads, which we determined with a dextran-rhodamine solution (hydrodynamic radius of ~5 nm) (Fig. 3c and Extended Data Fig. 8). Proteins that eluted early in the filtration assay (WDR1, GID8, CCS) mainly organized on the 10 nm scale. By contrast, proteins that we found in the squeezing regime (MVB12B, VGLL4, QRICH1, ILF3) exhibit $c_{in}\;/c_{out}\;\;$ increasing with the cutoff size, similar to established LLPS proteins (CIRBP, HNRNPA1) (Fig. 3d, e). This behavior suggests that the size of these assemblies is not sharply defined but spans across the sampled scale, matching the expectation for phase-separated droplets, which appear to assemble typically on the scale of ~100 nm.

We next sought to integrate our multiple proteomics experiments to improve predictions on which proteins are associated with phase-separated condensates. Existing predictors for proteins driving LLPS analyze published data to extract sequence features but suffer from a lack of comprehensive reference data. To this end, we trained a classifier, which learns to identify LLPS proteins by bagging an ensemble of linear discriminators and decision trees (Fig. 4a)^38–40^. We used the results from our filtration chromatography and dilution experiments as features. We additionally included coarsened sequence information on intrinsic disorder^41^, nucleic acid-binding^42–44^, and amino acid composition^43^. The performance of our predictor is assessed by the recall of known LLPS proteins with 5-fold cross-validation. While prediction using experiments alone already has an area under curve (AUC) of 0.86; our final predictor, including all features, reaches an AUC of 0.93 (Supplementary Table 2). This approach establishes an improvement over the state-of-the-art predictions of LLPS proteins by catGRANULE^45^ (AUC=0.84), Pscore^46^ (AUC=0.85), or PSAP^23^ (AUC=0.88). Notably, we gain sensitivity by a steeper early increase, which is arguably the most relevant regime for the prediction (Fig. 4b).

Finally, we sought to estimate what fraction of the proteome is organized into these liquid-like assemblies. To this end, we integrated our filtration chromatography and dilution experiments and developed a noise model based on replicates (Fig. 4c). BMCs exhibit a liquid-like behavior, different from membrane-bound organelles (MBOs) (Fig. 4d, e, Extended Data Fig. 9). About one quarter of the protein species exhibit behavior consistent with BMCs and is shifted beyond a 2% false discovery rate, including two thirds of the LLPS references (Fig. 4c). Additionally, only 12% of the detected proteins do not respond to the filtration and appear assembled smaller than the assayed scale. Correcting for MBOs, we conclude that at least 18% of the detected protein species are in BMCs (Fig. 4f). However, based on the spread of known BMCs into our noise model we consider this a very conservative lower bound (Fig. 4c). We expect a significant fraction of the remaining third of the protein species to be also organized via BMCs (Fig. 4f).

Previous studies have focused primarily on large (≥1 μm) BMCs, which are easily observed by microscopy. Nevertheless, recent studies have increasingly demonstrated smaller structures^34,35^. Our differential filtration and size exclusion studies on intact cytoplasm reveal that phase separation-prone proteins ubiquitously organize cytoplasm into mesoscale assemblies, which exhibit liquid-like deformability (Fig. 4g). Notably, these assemblies are more stable upon dilution than expected for assemblies formed through LLPS alone, and likely have less-dynamic, potentially solid-like core structures. Besides gelation or specific binding interactions, RNA may play a key role in this stabilization, as our data suggest that many RNAs are contained in BMCs. Furthermore, our findings illustrate the potential of proteomics data to enhance the prediction of LLPS proteins. Presumably, this can be effectively integrated with orthogonal approaches, such as those previously mentioned or those that analyze disordered sequences^47^.

It remains an exciting question how these tiny BMCs can exist without ripening into larger condensates, via coalescence or Ostwald ripening (the growth of large condensates at the expense of smaller ones). We speculate that mesoscale organization we have uncovered is highly dynamic, reflecting continuous assembly and disassembly. Such behavior can originate from associative polymers and their percolation^29,30^, or chemical activity^48^, but is also reminiscent of phase-separating systems in the vicinity of a critical point, as has been suggested for two-dimensional phase separation in the plasma membrane^49^.

## Methods

### Xenopus laevis egg extracts

#### Frog husbandry:

Mature Xenopus laevis females were purchased from Nasco/Xenopus1 and maintained by Laboratory Animal Resources at Princeton University. All animal procedures are approved under IACUC protocol 2070, reviewed in March 2023. Ovulation was induced with at least six-month rest intervals.

#### Egg collection:

*X. laevis* eggs were collected as previously described^51^ from wildtype females between 1 and 5 years old. Frogs were primed with pregnant mare serum gonadotropin (HOR-272, ProSpec-Tany TechnoGene Ltd) within two months before the experiment. At 16 h before egg collection, frogs were injected with 500 U of human chorionic gonadotropin (Sigma CG10) and kept at 16°C in Marc’s Modified Ringer’s^52^ (50 mM HEPES pH 7.8, 100 mM NaCl, 2 mM KCl, 1 mM MgCl_2_, and 2 mM CaCl_2_). We collected eggs the next day in MMR buffer and sorted out pre-activated ones for further use.

#### Extract preparation:

*X. laevis* egg extracts were prepared as previously described^53,54^. The eggs were dejellied in MMR with added L-cysteine (2 wt%, pH 7.8) and washed in CSF-XB buffer (100 mM KCl, 20 mM HEPES, 2 mM MgCl_2_, 0.1 mM CaCl_2_, 4 mM EGTA, pH 7.7). Eggs were collected into 3.5 ml centrifuge tubes (Beckmann) in the presence of cytochalasin D (Sigma C8273) and LPC protease inhibitor premix (leupeptin (Sigma L2884), pepstatin (Sigma P5318), chymostatin (Sigma C7268)). The surplus buffer is removed after a soft spin at 500 g for 1 min. Eggs are crushed and fractionated in a spin at 14400 g for 15 min. The cytoplasmic fraction is extracted using an 18G gauge needle. The extract was supplemented with 10 μg/mL cytochalasin D, 10 μg/mL LPC, 1 μM nocodazole (Sigma M1404), and 50 mM sucrose. All used drugs were dissolved in DMSO, resulting in a total DMSO concentration of <2.5 permille. We prefiltered extracts through a 6 μm polyether mesh filter to remove residual debris and stored them on ice for further use.

### Preparation spin control experiment

Immediately after the preparation spin, the centrifuge tube containing the spin-crushed and sedimented eggs is shock-frozen in liquid nitrogen at 77 K. The extract section, excluding lipid and yolk/debris sections, is cut out and cut in halves using a razor blade.

### Filtration experiments

Filtrations are performed in 2 ml tubes in a tabletop centrifuge, using 3D printed filter holders^55^. The design files are available on our GitHub page:

https://github.com/wuhrlab/3DFilterHolderDesigns.

We used the Hubs platform (https://www.hubs.com/) to print the holders using either Standard Resin (SLA) or Dental resin (SLA) materials at 20% infill rate, 50 μm layer height. We use polyethersulfone membranes (Sterlitech, PES00347100, PES0147100, PES0247100, PES0847100) in the spin filtration setup and the in vitro assay, and cellulose acetate membranes (Sterlitech, C080A047A, C300A047A, CA1247100, CA0247100) in the gravity flow setup. Membranes were wetted with methanol and washed with CSF-XB buffer. After excess buffer removal, the sample chamber was flushed twice with 50 μl cell extract.

#### Filtration chromatography experiments:

50 μL (167 μL) of the cytoplasmic extract was loaded and spun at the respective speed (at 100 g (30 g) for early timepoints, at 1000 g (300 g) for late timepoints) in turns of 4 min until approximately 15 μL of filtrate was collected. To avoid material build-up or cake formation, samples are spun at fixed angle (45°) and we stirred the sample chamber after each turn. The input sample (~10 μL) was collected before the spin.

#### Dilution experiments:

30 min before the spin filtration process, the extracts were diluted in CSF-XB buffer in a dilution series to a factor of 1.2, 1.44, and 2.

#### RNase experiments:

1 U/μl of RNase I (Ambion) was added to the solutions 45 min before the filtration.

#### Gravity flow experiments:

200 μL samples were loaded and placed in a box providing a humid atmosphere until sufficient flowthrough accumulated. The input chamber was stirred occasionally to avoid clogging.

### Sedimentation assay

200 μl of extract were spun in 5×20mm polypropylene tubes (Beckman Coulter) at 200 krcf for 15 min (30 min), using a TLS55 swinging bucket with tube adaptors in an Optima TLX ultracentrifuge (Beckman Coulter). Samples were flash frozen in liquid Nitrogen and cut into halves.

### Expression of GFP-fusion proteins

The Gateway entry plasmids of desired proteins were retrieved from *Xenopus laevis* ORFeome^56^. The destination vector carrying an EGFP sequence-TEV site-S-tag was purchased from Addgene (pCSF107mT-GATEWAY-3’-LAP tag, plasmid #67618). For the Gateway LR cloning reaction, the entry plasmid, the destination plasmid, and the Gateway LR clonase II enzyme mix (Invitrogen 11791) were combined at the ratios recommended in the manufacture protocol. After the reaction, the expression cloned vector was purified, then linearized using restriction enzymes, which were chosen so that the region of protein of interest was protected. The linearized plasmids were in-vitro transcribed using the mMESSAGE mMACHINE SP6 kit (Invitrogen AM1340) supplemented with a 7-methyl guanosine cap protected on the 5’ end terminal, and a poly(A) tail (NEB M0276). Finally, RNA products were purified using Trizol LS reagent (Invitrogen 10296010), then resuspended in nuclease-free water at ~1 μg/μL in the final RNA concentration. The RNA solution was added in a volume ratio of 1:100 to the extracts.

### Size exclusion assay

Size exclusion chromatography beads (GE, Sephacryl High Resolution, S-200/300/400/500, 17-584-10/17-599-99/17060999/170613-10; molecular size cutoffs 400 kDa/2 MDa/9 MDa/100 MDa) were washed in CSF-XB buffer and equilibrated in three rounds of sedimentation, supernatant removal, 1:5-add-up in plain cell extract and waiting times of about 10 min. After being finally added to the labeled cell extracts, we waited at least 15 min before imaging. The samples were enclosed in mineral oil to prevent evaporation (Sigma M5904). For calibration, we added the beads to a 70kDa-Dextran-rhodamine-B-isothiocyanate (Sigma, R9379, approx. 5 nm) solution in CSF-XB buffer. Sizes were estimated from molecular weights using Zetasizer (Malvern Panalytical).

#### Image analysis:

Line profiles of beads and surrounding solution were measured manually in ImageJ/Fiji^57,58^. Raw image intensities were corrected for the detector background to make them proportional to concentrations. An estimation of the accessible volume for each bead type was measured by the intensity ratio for the dextran solution. The GFP intensities measured inside the bead were adjusted by this factor to calculate the concentration ratio.

### Microscopy

Confocal images of labeled cytoplasmic extracts were taken in glass-bottom well-plates (Cellvis) on a Nikon A1 laser scanning confocal microscope using 60x and 20x oil immersion objectives. Images of the in vitro assay were taken on a Nikon spinning disc confocal microscope with a 100x oil immersion objective. Fluorescence correlation microscopy (FCS) was performed with an oil immersion objective (Plan Apo 60X/1.4 numerical aperture, Nikon) using an FCS Upgrade Kit for Laser Scanning Microscopes (PicoQuant). FCS measurements were performed using the SymPhoTime Software (PicoQuant).

### MS sample preparation and analysis

#### Low complexity samples:

Preparation spin control, sedimentation assay, RNAse treatment, gravity flow, filtration data used for fitting the model, and filtration at 200 nm. Labeled using TMT-10plex and TMTpro 16plex (Thermo Fisher Scientific), analyzed by TMT-MS3^59^ and TMTproC (RNAse treatment). See Supplementary Table 3 for detailed tag assignment to channels.

#### High complexity samples:

Filtration chromatography at 30 nm and 100 nm. Labeled using TMT-10plex, analyzed by TMTc+^60^. Filtration of diluted cytoplasm. Labeled using TMT-10plex and TMTpro-16plex (Thermo Fisher Scientific), analyzed by TMTproC^20^. See Supplementary Table 3 for detailed tag assignment to channels.

#### Sample preparation:

Samples were prepared mostly as previously described^61^. Lysates were collected in 100 mM HEPES pH 7.2 and proteins were denatured by adding 2% SDS at volumes ~100 μL. Concentrations were determined by Bicinchoninic acid (BCA) assay (Pierce) and similar amounts underwent further processing. All conditions for a multiplex were prepared in the same batch. To reduce disulfides, Dithiothreitol (DTT) (500 mM in water) was added to a final concentration of 5 mM (20 min, 60°C). Samples were cooled to RT, and cysteines were alkylated by the addition of N-ethyl maleimide (NEM, 1 M in acetonitrile) to a final concentration of 20 mM followed by incubation for 20 min at RT. 10 mM DTT (500 mM stock, water) was added at RT for 10 min to quench any remaining NEM. A methanol-chloroform precipitation was performed for protein clean-up, and the collected protein pellets were allowed to air dry. Samples were taken up in 6 M guanidine chloride in 200 mM EPPS pH 8.5. Subsequently, the samples were diluted to 2 M guanidine chloride in 200 mM EPPS pH 8.5 for overnight digestion with 20 ng/μL Lys-C (Wako) at RT. The samples were further diluted to 0.5 mM guanidine chloride in 200 mM EPPS pH 8.5 and then digested with 20 ng/μL Lys-C and 10 ng/μL trypsin (Promega) at 37°C overnight. For the samples of the sedimentation assay and the model fit, methanol-chloroform precipitation was replaced by SP3 magnetic bead (SpeedBead Magnetic Carboxylate, Thermo scientific 45~/65152105050250) cleanup^62^.

The digested samples were dried using a vacuum evaporator at RT and taken up in 200 mM EPPS pH 8.0. To equalize channel loading, same protein masses for each condition in a multiplexed sample were labeled with tandem mass tags. The total mass per sample was ~20 μg/~200 μg for low complexity/high complexity samples respectively. TMT/TMTpro samples were labeled for 2 hours at RT. Labeled samples were quenched by adding 0.5% hydroxylamine to the solution. Samples from all conditions were combined into one tube, acidified to pH < 2 with phosphoric acid (HPLC grade, Sigma) and cleared by ultracentrifugation at 100,000 g at 4°C for 1 hour in polycarbonate tubes (Beckman Coulter, 343775) in a TLA-100 rotor. Supernatants were dried using a vacuum evaporator at RT. For a low complexity sample, dry samples were taken up in HPLC-grade water and stage-tipped for desalting^63^ and resuspended in 1% formic acid (FA) to 1 μg/μL for mass spectrometry analysis. For high complexity samples, the supernatant was sonicated for 10 minutes and then fractionated by medium pH reverse-phase HPLC (Zorbax 300Extend C18, 4.6 × 250 mm column, Agilent) with 10 mM ammonium bicarbonate, pH 8.0, using 5% acetonitrile for 17 minutes followed by an acetonitrile gradient from 5% to 30%. Fractions were collected starting at minute 17 with a flow rate of 0.5 mL/min into a 96 well-plate every 38 seconds. These fractions were pooled into 24 fractions by alternating the wells in the plate^64^. Each fraction was dried and resuspended in 100 μL of HPLC water. Fractions were acidified to pH <2 with HPLC-grade trifluoroacetic acid, and stage-tipping was performed to desalt the samples. For LC-MS analysis, samples were resuspended to 1 μg/μL in 1% FA and HPLC-grade water, and ~1 μg of peptides were analyzed per 1 hour run time. Quality of the sample preparation was controlled by checking the labeling degree, channel loading, content of cysteine-containing peptides, and missed cleavages in a single-shot MS3 analysis.

#### MS analysis:

Approximately 1–3 μg of the sample was analyzed by LC-MS. LC-MS experiments were performed with an nLC-1200 HPLC (Thermo Fisher Scientific) coupled to an Orbitrap Fusion Lumos (Thermo Fisher Scientific). Injected volumes were in the range of 1–4 μl. For each run, peptides were separated on an Aurora Series emitter column (25 cm x 75 μm ID, 1.6 μm C18) (ionopticks, Australia), held at 60°C during separation by an in-house built column oven. Separation was achieved by applying a 12% to 35% acetonitrile gradient in 0.125% formic acid and 2% DMSO over 90 min for fractionated samples and 180 min for unfractionated samples at 350 nL/min at 60°C. Electrospray ionization was enabled by applying a voltage of 2.6 kV through a MicroTee at the inlet of the microcapillary column. As indicated in each proteomics experiment, we used the Orbitrap Fusion Lumos with a TMT-MS3^59^, TMTc+^60^, or TMTproC^20^ as previously described.

### Reference databases

If not stated otherwise, all annotations are from Uniprot^43^.

#### Complexes and organelles:

Our reference group for large ‘complexes’ includes proteins from ribosome, proteasome, the vault complex, Arp2/3, RNA polymerase II core complex, PCNA-DNA polymerase delta complex, and gamma-tubulin ring complex (the latter four from CORUM^65^). In the group ‘Mitochondrion’, we exclude proteins with promiscuous subcellular location annotations. The reference group ‘BMC-associated proteins’ is the union of the categories’ client’ and ‘regulator’ in DrLLPS^24^. Organelles in DrLLPS that had an abundance of client and regulator annotations were further refined using additional databases: ‘Stress granule’ and ‘P-body’ by the RNPgranule database^66^, and ‘Nucleolus’, ‘Postsynaptic density’, ‘Centrosome/Spindle pole body’, and ‘PML nuclear body’ by UniProt subcellular location. For the estimation of the fraction of proteins in membrane-bound organelles, we use QuickGO^44^ and restrict to ‘UniProtKB’(swiss-prot) and ‘located in’ membrane-bound organelles. ‘Small’ proteins were located around the origin in our filtration experiments within the bounds of our null-model. Transmembrane helices were predicted by Krogh’s algorithm^67^.

#### LLPS database:

To create a comprehensive, high-confidence database of known LLPS proteins with minimal personal curation bias, we follow the approach of merging several published databases^15^: PhasePro^22^, DrLLPS^24^, PhaSepDB^25^, LLPSDB^26^, and extend this set by the reference list for the PSAP predictor^23^. We furthermore include proteins assigned ‘candidate’ in PhasePro and the updated annotation ‘PS-self’ in PhaSepDB version 2 that indicates de-novo phase separation without partners. We define the ‘consensus level’ as the number of databases in which a protein is found; throughout the manuscript we use consensus level 4, except Fig. 2 (level 3) and Extended Data Fig. 4 (level 0). Overall, LLPS proteins with higher consensus level showed more significant shifts. This may reflect the increasing curation quality but may also be due to system specific or partner dependent phase separation.

### Predictor learning

We train our predictor for phase separating proteins in MATLAB (MathWorks), using the function fitcensemble for ensemble classification. Class one is proteins from our LLPS database and class two is the rest of the proteome. We use only proteins identified in both the filtration chromatography and the dilution experiments (N~4000) and thus have no missing values. The features are the filtrate concentrations relative to the input in the different conditions (filtration chromatography: 30 nm and 100 nm at $T_{1}\;$ and $T_{2}$. Diluted filtration: 100 nm diluted 1x (undiluted)/1.2x/1.4x/2x). We include features for intrinsically disordered regions determined by Espritz^41^ (fraction of amino acids (aa) in IDRs, number of IDRs of >50 aa/>30 aa/any-length, setting: disprot, BestSw). We include features on DNA binding^43^, RNA binding (QuickGo^44^) and RNA-binding domains^42^. Following van Mierlo et al.^23^, we also include the sequence fractions of Glycine, Cysteine, Leucine, Isoleucine, as well as the content of aliphatic and aromatic residues. We use the method ‘Bag’ to train ensembles with 500 learners of decision trees and linear discriminators^38–40^. The trees are restricted to a minimum number of 32 proteins per leaf and have a maximum number of splits equaling three quarters the number of used features. We train N=600 ensembles on partitions of the data using 80% of class 1 (N1~40) and 10% of class 2 (N2~400). Thus, we obtain each protein’s cross-validated score by the median score of these ensembles, excluding any runs where it was used in the training. When trained separately on experiment ($s_{exp}$) and sequence features ($s_{seq}$) and combined by the Euclidean distance, $s=\sqrt[{2}]{{\left(\max\left(s_{exp}\right)-s_{exp}\right)}^{2}+{\left(\max\left(s_{seq}\right)-s_{seq}\right)}^{2}}$, the final score s shows a slightly better performance (AUC for recall LLPS 0.93 vs 0.91). Likely, this can be accounted to the relatively small training sets and that there are more sequence features than experimental features. The PSAP predictor could not be plotted, as its published output does not contain cross-validated results of the training set.

### Data processing

#### Data exclusion:

For all the experiments reported in the manuscript, no data was excluded.

#### Normalization:

Filtration data are compared using their fold changes $FC=c/c_{0}$, where the concentrations c are assumed proportional to the MS signal sum of peptides’ reporter ions (MS3 method) or complementary ions (TMTc method). The FCs were normalized separately for each experiment to the 0.95 quantile of each experiment to account for loading imbalance in the TMT channels. Proteins with the highest FCs pass the filter medium fastest and represent unhindered flowthrough.

#### Squeezing score:

The upper edge close to the identity line of the scatter data is determined by fitting a line and taking the top four percent of points in bins along the line. Another line is fit through these points and the squeezing score is the orthogonal distance to it.

#### Null model:

We create an empirical null model for the measurement noise. We match replicates with a Pearson correlation larger than 0.8 from 36 early elution conditions and fit a line through each of the resulting 359 sample pairs in log_2_-space. The resulting histogram of residues (N=393k) serves as an estimate for the errors.

#### Fraction of BMCs:

To estimate the fraction of the proteome that is organized in liquid assemblies, we further constrain this by fitting the line through large complexes and proteins with transmembrane domains^67,68^. We average the shifts in the 30 nm and 100 nm filtration chromatography and the 1.2x and 1.44x dilution experiments to quantify the ‘liquid-like behavior’. We identify proteins in BMCs beyond a 2% false discovery rate of the null model, omitting any proteins with membrane annotation.

### Mass spectrometry data analysis

MS data analysis was performed essentially as previously described^69^, using the Gygi Lab software platform (GFY Core Version 3.8) licensed through Harvard University. The MS data in the Thermo RAW format was converted to mzXML format and erroneous assignments of peptide ion charge state and monoisotopic m/z were corrected^70^. Monoisotopic mass detection was supported by Monocle^71^. ReAdW.exe was modified to include signal to noise (S/N) ratios for each peak during file format conversion (http://sashimi.svn.sourceforge.net/viewvc/sashimi/). Assignments of MS2 spectra were performed by the SEQUEST algorithm^72^, searching against a combined database made of (i) the *X. laevis* v9.2 genome assembly (Xenbase^73^ (RRID:SCR_003280)), (ii) common contaminants like human keratins and digestion enzymes, (iii) the reverse protein sequences of the target ((i) and (ii)) as a decoy. Searches were performed using a precursor ion tolerance of 20 ppm and a product ion tolerance of 1 Da or 0.02 Da for MS3 or TMT(pro)c methods respectively. Both peptide termini were required to be consistent with Lys-C/Trypsin digest specificity, allowing one missed cleavage. Static modifications included TMT/TMTpro tags on lysine residues and peptide N-termini (+229.162932 Da/+304.2071 Da), and NEM on cysteine residues (+125.047679 Da). Up to three differential modifications included oxidating of methionine residues (+15.99492 Da) and water addition to NEM on cysteines (+18.0105 Da). An MS2 spectral assignment false discovery rate of less than 1% was achieved by applying the target-decoy database search strategy^74^. Filtering was performed using a linear discrimination analysis method to create one combined filter parameter from the following z-scored peptide ion and MS2 spectra properties: SEQUEST parameters XCorr and Diff_Seq_dCN, missed cleavages, adjusted ppm, peptide length, fraction of ions Matched, and charge state. Forward peptides within 3 standard deviation of the theoretical m/z of the precursor were used as positive training set. All reverse peptides were used as negative training set. Linear discrimination scores were used to sort peptides with at least 7 residues and to filter with a cutoff of 1% false discovery rate based on the decoy database^70^. Each search was software-recalibrated to alleviate any systematic mass error dependent on peptide elution time or observed m/z. All ions in the full MS1 spectra were first adjusted. A representative subset of peptides was selected using those above the median XCorr and within one standard deviation of the global mass error. The mass errors of this subset were then fit to each parameter using LOESS regression. The m/z of every ion in MS1 spectra was then adjusted by the error predicted by interpolating the values of the nearest data points in the regression model. Adjustments for each of the two parameters were done iteratively. MS2 spectra were then calibrated in a similar manner. Mass errors were calculated from matched peptide fragment ions within two standard deviations of the global mass error and above the upper quartile of intensity. Mass errors were fitted to each parameter using LOESS regression and the m/z for every ion in MS2 spectra was adjusted as above. Peptides that matched multiple proteins were assigned to the proteins with the greatest number of unique peptides. TMT-MS3^59^, TMTc+^60^, or TMTproC^20^ data were analyzed as previously described (MATLAB module available on github: https://github.com/wuhrlab/TMTProC).

The mass spectrometry proteomics data have been deposited to the ProteomeXchange Consortium via the PRIDE^75^ partner repository with the dataset identifier PXD029879.

### RNA-seq library preparation and sequencing

Procedures for RNA extraction from input, low and high filtered samples were similar to protein samples preprocessing for mass spectrometry, except that the samples were immediately lysed in TRIZOL, frozen in liquid nitrogen, and stored at −80°C. Three biological replicates were collected in total. RNA extraction was performed using phenol chloroform precipitation and purified with the RNA Clean & Concentrator kit (Zymo) following the manufacturer’s instructions, including a 10-minute DNase treatment in column. The quality of the RNA was assessed using NanoDrop (Thermo Fisher Scientific), Qubit (Invitrogen), and TapeStation system (Agilent Biotechnologies), and only samples with high quality (RNA integrity number ≥ 7.0) were used to prepare libraries on an Apollo 324 system with the PrepX RNA-seq protocol (Takara Bio) and a ribo-depletion step (RiboCop rRNA, Lexogen). Paired-end (300 cycles total) sequencing was performed on an NovaSeq SP 100nt Flowcell v1.5 (Illumina) at the Genomics Core Facility of Princeton University with a read depth of 30–90 million reads per sample.

### Bioinformatic analysis of sequencing results

The RNA-Seq reads were first assessed using FastQC (0.10.0)^76^ (http://www.bioinformatics.babraham.ac.uk/projects/fastqc/) and TrimGalore (0.6.10)^77^ for adapter removal and quality control (http://www.bioinformatics.babraham.ac.uk/projects/trim_galore/). The processed reads were then aligned to the *X. laevis* v10.1 reference genome (Xenbase, http://www.xenbase.org/entry/) using STAR (v2.7.10a) with the option “--quantMode GeneCounts”^78^. The output files were imported into R (v3.5.1). Fold changes between $T_{1}$ and $T_{2}$ filtration vs input samples were calculated, after performing median of ratios normalization and rlog transformation using DESeq2 (v1.32.0)^79^. Samples similarity was assessed using hierarchical clustering with both Euclidean and Pearson distances. Functional enrichment analysis was done using the STRING database^50^ and its reported false discovery rates determined by Bonferroni corrected Kolmogorov-Smirnoff tests. The category “Development-related” was built by text-mining all enrichments for the terms “morphogenesis”, “development”, “differentiation”, “fate” or “growth”. All RNA-seq datasets are deposited in the National Center for Biotechnology Information’s (NCBI) Gene Expression Omnibus (GEO) with the GEO accession number GSE232651.

### Randomization and Blinding

No randomization was performed. Randomization is not relevant to this study for comparisons within one set of isoboaric tags in multiplexed proteomics studies. Data collection and analysis were not performed blind to the conditions of the experiments. Proteomics or transcriptomics samples underwent the same workflow during which they were indistinguishable.

### Data Availability

Assignments of mass spectrometry spectra were searched against the X. laevis v9.2 genome assembly (Xenbase (RRID:SCR_003280)^73^. LLPS database was sourced from PhasePro^22^, PhaSepDB^25^, DrLLPS^24^, LLPSDB^26^, and the references from the PSAP predictor^23^; protein complex data from CORUM^65^. Protein nucleic acid binding was sourced from Uniprot^43^, QuickGO^44^, and Castello et. al.^42^. Organelle data was sourced from DrLLPS^24^, Uniprot^43^, and RNPgranuleDB^66^. Predictions of catGRANULE^45^, Pscore^46^ or PSAP^23^ were reevaluated and plotted. Intrinsically disordered regions, transmembrane helix annotations, and enrichment analysis were generated from Espritz^41^, Krogh et.al.^67^, and STRING^50^ respectively. Housekeeping gene and L-body annotations were derived from Eisenberg et. al.^31^ and Neil et. al.^33^. The mass spectrometry proteomics data have been deposited to the ProteomeXchange Consortium via the PRIDE partner repository with the dataset identifier PXD029879. The raw sequencing data and gene expression matrices have been deposited to the National Center for Biotechnology Information’s (NCBI) Gene Expression Omnibus (GEO) with the GEO Series accession number GSE232651. All other data are available from the corresponding authors upon reasonable request. Source data are provided with this study in Source Data and Supplementary Table 3.

### Code availability

Custom code is available from the corresponding authors upon reasonable request. Code for the analysis of TMTproC data is available on GitHub: https://github.com/wuhrlab/TMTProC.

### Statistics and Reproducibility

**Extended Data Fig. 1:**
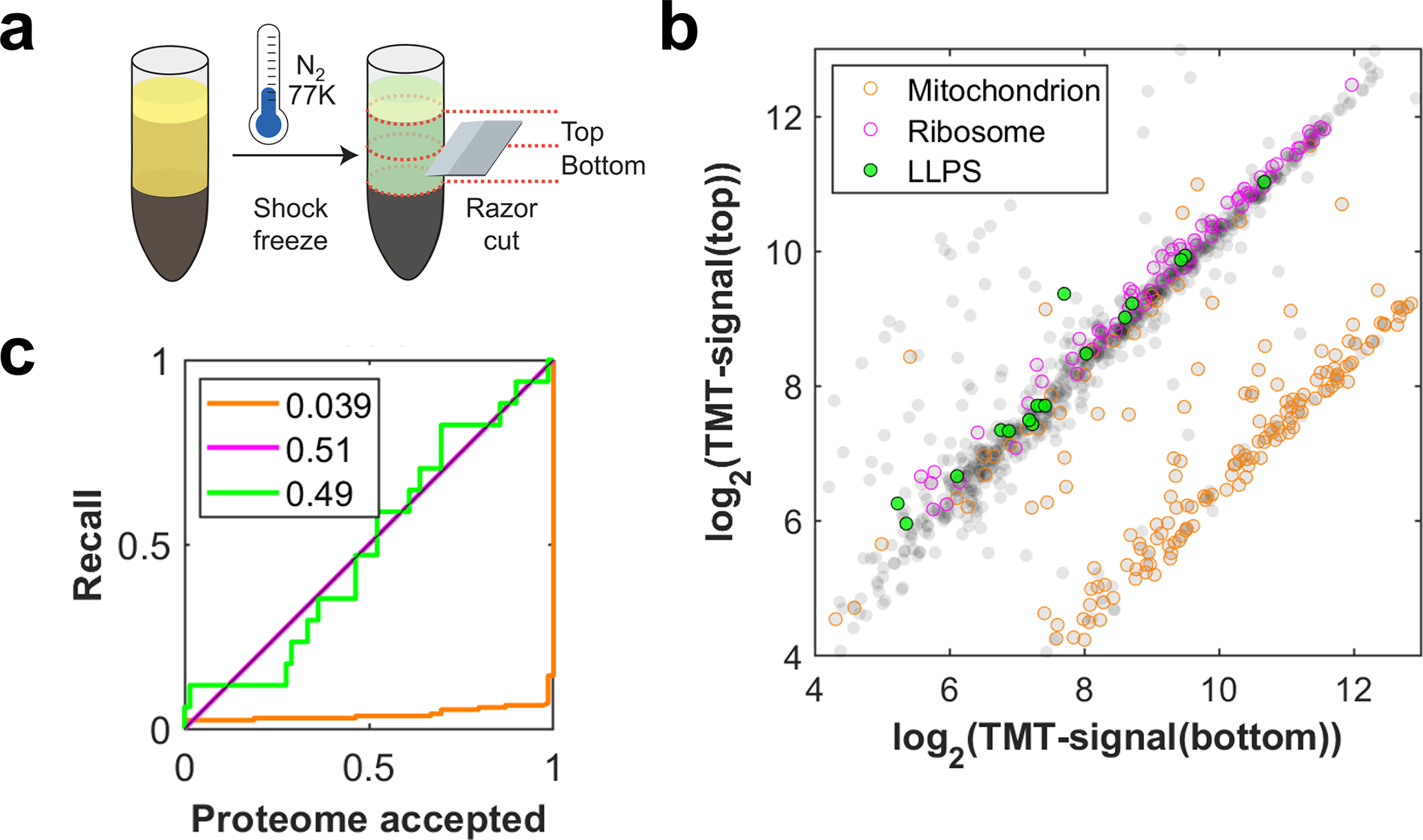
Sedimentation in extract preparation spin is negligible. **a,** Schematic illustrating the sample collection. After 16 min of the preparation spin (14400 krcf), the tube is shock frozen in liquid nitrogen and subsequently cut into a top and a bottom section with a razor blade. **b,** Comparison of the two halves of the extract. While mitochondrial proteins (orange) are shifted to the bottom, indicating sedimentation, the bulk part of the proteome, including both ribosomal (magenta) and LLPS proteins (green), stays unchanged. Scatter plot of raw TMT signals. N=1 biological sample. **c,** Receiver operator characteristics on the signal ratio top to bottom of the data in (b). Source numerical data and proteomics data are provided in Source Data and Table 3.

**Extended Data Fig. 2:**
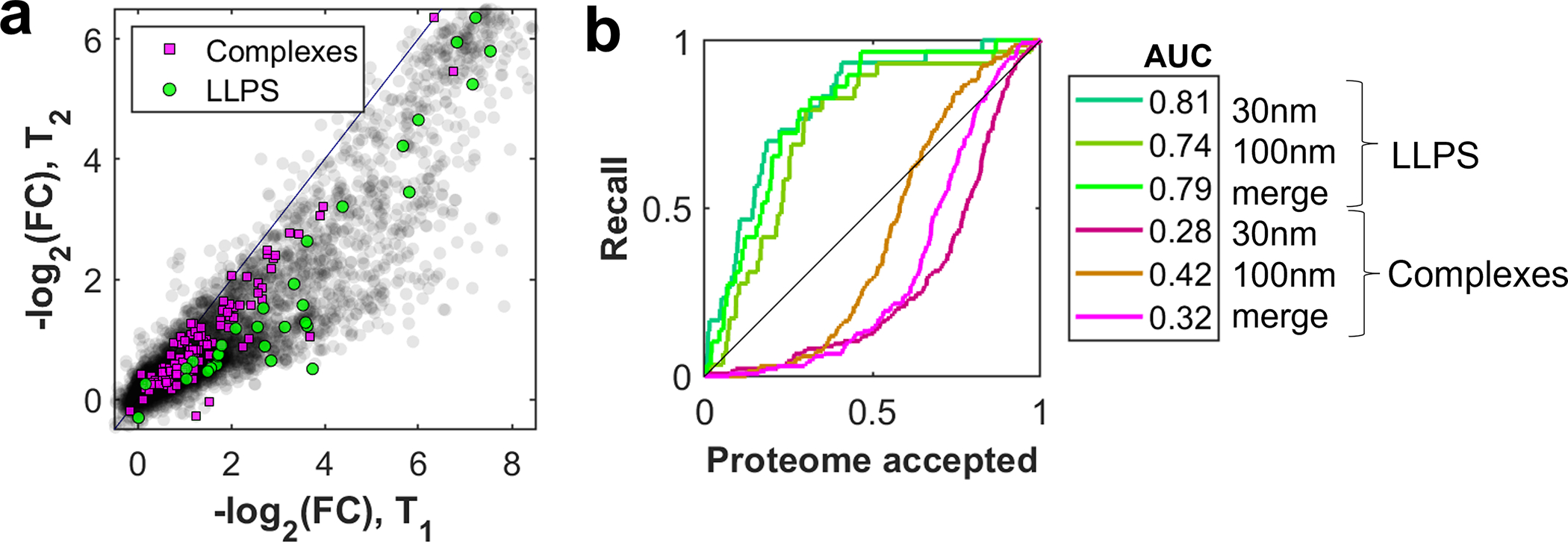
Filtration at pore size 100 nm. **a,** Executing the experiment presented in Fig. 1c with a larger pore size ($d_{pore}=100\;nm$) yields qualitatively similar results. However, there are quantitative changes, and, as expected, the overall permeation is higher. FCs were normalized separately for each experiment to their 0.95 quantile, representing freely passing proteins. N=1 biological sample. **b,** The receiver operating characteristic for LLPS proteins is best for the 30 nm filtration (AUC=0.81). Notably, the separation of the LLPS and complexes groups is weaker in the 100 nm condition. Source numerical data and proteomics data are provided in Source Data and Supplementary Table 3.

**Extended Data Fig. 3:**
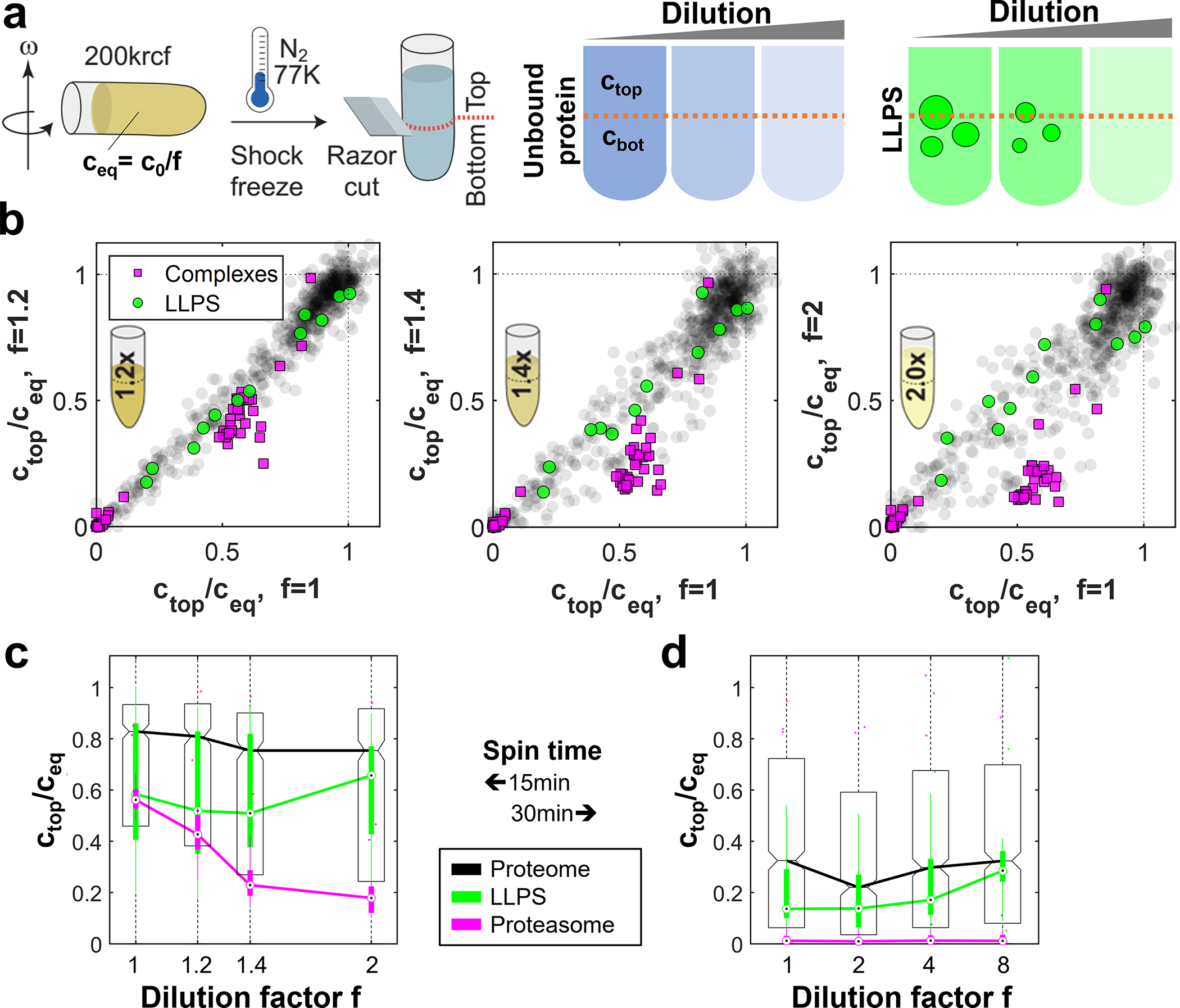
Sedimentation of diluted cytoplasm suggests partial dissolution of condensates. **a,** Schematic of sedimentation assay. Extract is diluted by a factor f and centrifuged in a hard spin; the top and bottom part are analyzed by mass-spectrometry. For an unbound protein, the top and bottom part have equal concentrations independent of dilution, $c_{top}=c_{bot}=\frac{c_{0}}{f}≔c_{eq}$. By contrast, phase separated proteins sediment in assemblies and the concentration in the bottom part is higher unless they fully dissolve upon dilution. **b,** Scatter plots of sedimentation data for different dilution conditions (f=1.2/1.4/2.0) against the undiluted case (f=1). Concentrations are normalized to $c_{eq}$, derived from the 20% least sedimenting proteins. At the chosen timepoint (200 krcf, 15 min), the ribosome is fully sedimented (magenta, bottom left corner), while the proteasome (magenta cluster, middle) is not. The proteasome indicates facilitated sedimentation for the diluted conditions, however LLPS proteins (green) still exhibit similar $c_{top}/c_{eq}$. N=1 biological sample. **c,** Box-plot representation of the data in (b). In all tested dilutions, most LLPS proteins are observed to sediment. While their median starting point suggests sedimentation similar to the proteasome, $c_{top}/c_{eq}\;$ remains on a similar level, whereas the proteasome sediments further. This (together with (d)) may be explained by the counteracting effects of sedimentation and dissolution. N=1 biological sample, number of proteins per group: 788 (proteome), 35 (proteasome), 11 (LLPS). **d,** At longer centrifugation times (200 krcf, 30 min), an overall higher sedimentation is observed, along with a trend towards equilibration upon higher dilution. Importantly, LLPS proteins are sedimenting even up to 8-fold dilution. N=1 biological sample, number of proteins per group: 844 (proteome), 19 (proteasome), 11 (LLPS). Boxplots (c, d) display data distribution with the center as the median, box limits as quartiles, and whiskers to non-outlier extremes. Source numerical data and proteomics data are provided in Source Data and Supplementary Table 3.

**Extended Data Fig. 4:**
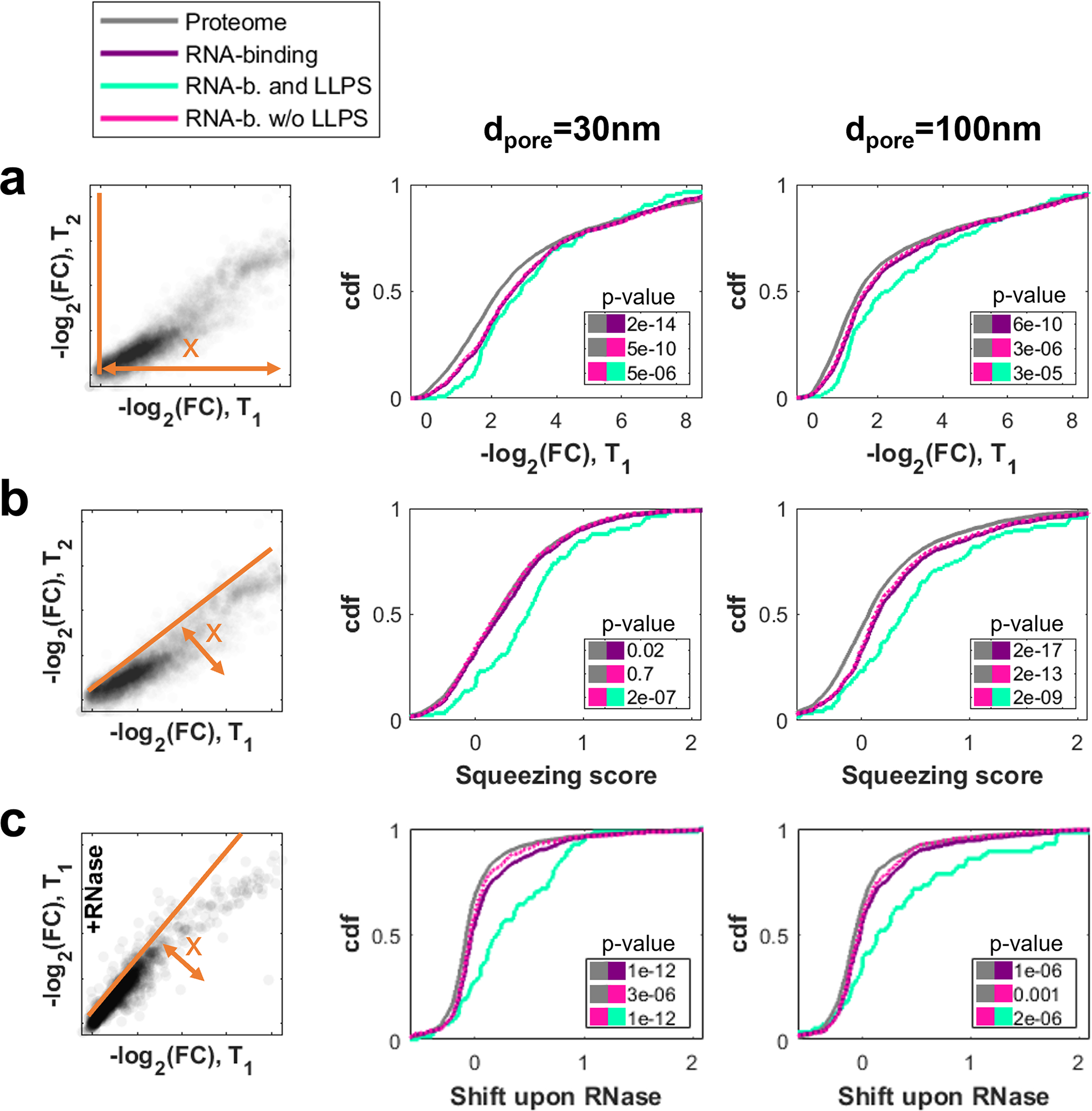
RNA binding proteins elute later, especially when part of liquid assemblies. Comparison of the flowthrough behavior of the proteome (gray) with RBPs (purple), grouped by their LLPS annotation (w: aqua, w/o: pink). Left column depicts which metric is used in the cumulative histograms for 30nm (middle) and 100 nm (right) pore diameters. The insets list the p-values (two-sided ks-test) between the groups color-coded by the marker. **a,** In the filtration experiment (see Fig. 1), RBPs exhibit higher retention than the bulk proteome. Notably, only few RBPs pass the filters unhindered. These observations are more pronounced for the LLPS subgroup. FCs were normalized separately for each experiment to their 0.95 quantile. N=1 biological sample. Proteins per group 3922 (proteome), 941 (RNA-binding), 116 (RNA-b. and LLPS), 825 (RNA-b. w/o LLPS). **b,** The squeezing behavior of most RBPs is slightly greater than that of the average protein. However, the LLPS subgroup shows a much greater shift. N=1 biological sample. Proteins per group 3922 (proteome), 941 (RNA-binding), 116 (RNA-b. and LLPS), 825 (RNA-b. w/o LLPS). **c,** Filtration of RNase-treated cytoplasm facilitated the overall flowthrough of RBPs. Again, the effect was much stronger on the LLPS subgroup, suggesting disruption of their assemblies. N=1 biological sample. Proteins per group 1802 (proteome), 489 (RNA-binding), 69 (RNA-b. and LLPS), 420 (RNA-b. w/o LLPS). Source numerical data and proteomics data are provided in Source Data and Supplementary Table 3.

**Figure 1:**
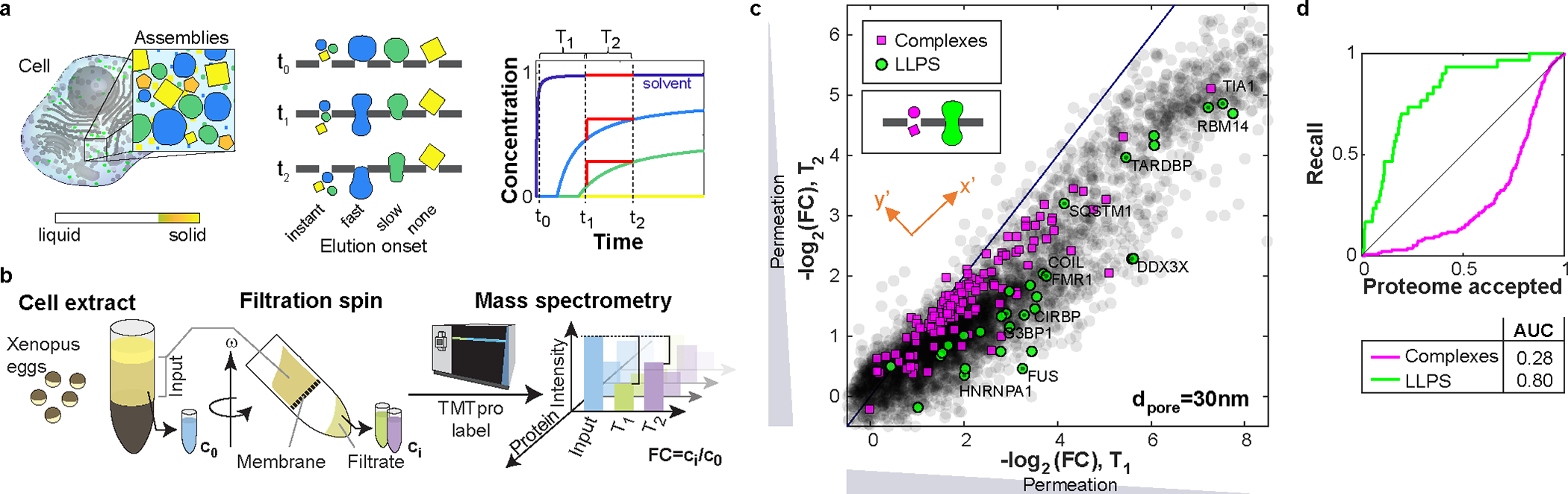
Proteomics of filtrated cytoplasm reveals organization in liquid-like assemblies. **a,** Illustration of the filtration process. For larger assemblies, squeezing through pores enables passage, where more liquid assemblies pass faster. Right: Sampling protein concentrations throughout the process (red lines) informs about the different elution behaviors. **b,** Experimental outline. We spun undiluted cytoplasm from frog eggs through filters and analyzed the filtrate in comparison to the input by mass spectrometry. For each protein, we obtain the concentration fold change between input and filtrate $FC=c/c_{0}$ via quantitative proteomics. $T_{i}$ denotes the time interval of the sampling $\left[t_{i-1},t_{i}\right]$). FCs were normalized separately for each experiment to their 0.95 quantile, representing freely passing proteins. **c,** Scatter plot of protein permeation later ($T_{2}$) versus earlier ($T_{1}$) in the process. Close to the identity line (blue), the permeation is almost unchanged -corresponding to the flat part of the curves in (a). Below the identity line permeation is increased in $T_{2}$ -as expected for a steep curve section in (a). Proteins established to form LLPS (green), or large complexes (magenta) exhibit distinguishable behaviors, matching the expectation (inset schematic). N=1 biological sample. **d,** We rank order the proteome along an axis orthogonal to the upper edge of the data close to the identity line (y’ in orange inset in (c)). The receiver operating characteristic (ROC) plots the true positive rate (recall) against the false positive rate (proteome accepted). A perfect classifier’s curve would pass through the top-left corner, whereas a diagonal line indicates randomness. LLPS proteins are recalled against y’ preferentially, while complexes are underrepresented. This distinction is quantified by the area under the curve (AUC). Source numerical and proteomics data are provided in Source Data and Supplementary Table 3. N=1 biological sample.

**Figure 2:**
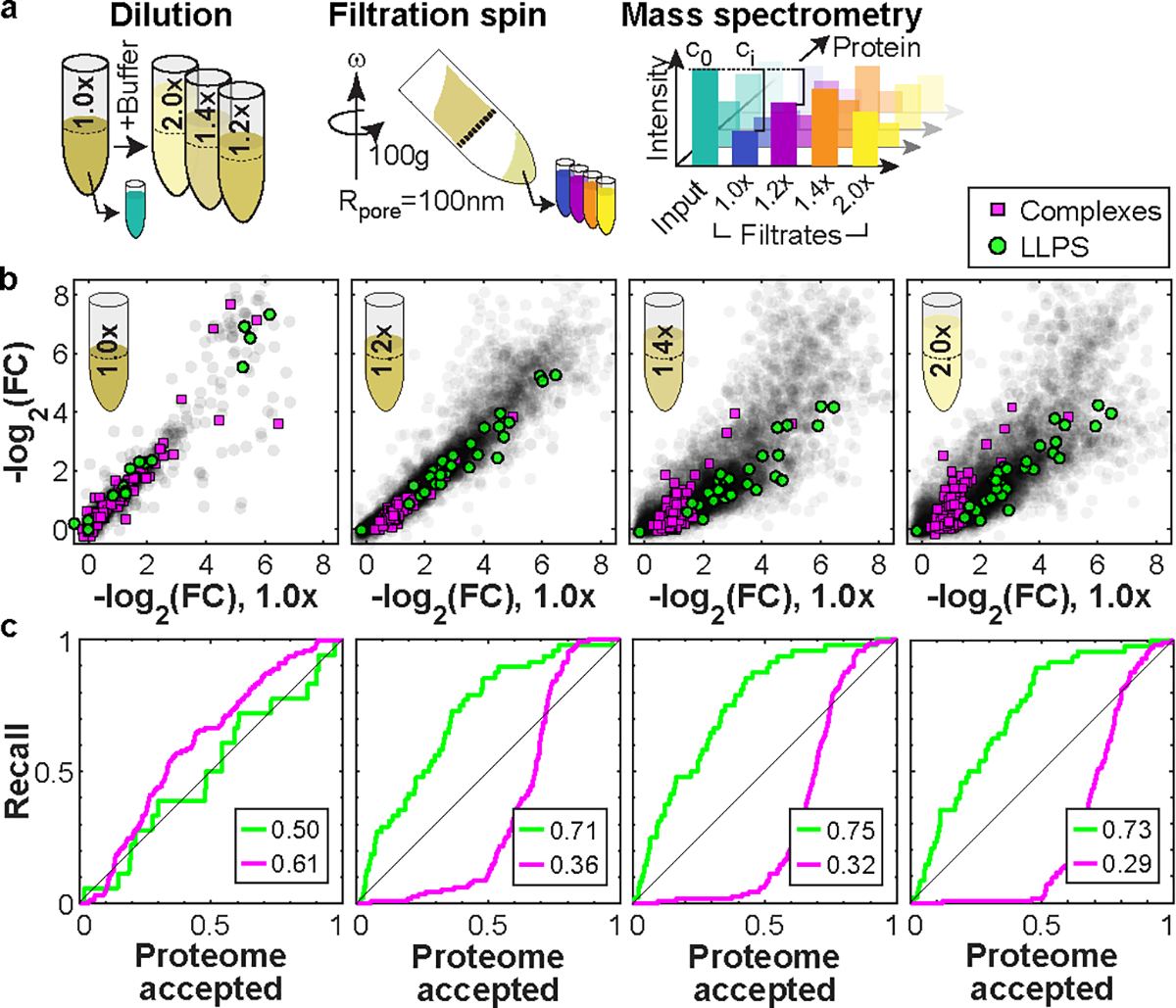
Liquid-like assemblies are sensitive to moderate dilution. **a,** We performed the cytoplasmic filtration experiment with 100 nm pores and compared protein retention with lysate diluted by various amounts. The concentrations of the filtrates relative to the input are measured by mass spectrometry. **b,** Experiment of filtration of diluted lysate reveals that LLPS proteins (green) seem to disassemble at dilutions larger than 1.2x. FCs were normalized separately for each experiment to their 0.95 quantile, representing freely passing proteins. N=1 biological sample. **c,** Receiver operating characteristics to the scatter plots above along an axis orthogonal to the upper edge of the data, with the AUCs displayed in the boxes. Source numerical and proteomics data are provided in Source Data and Supplementary Table 3.

Proteomics experiments on filtration chromatography and dilution presented in Figures 1 and 2 and Extended Data Figures 1, 2, 3, 4, 6, and 9 were performed on n=1 biological sample each. This way, the number of conditions compared in a multiplex could be maximized. Proteomics replicates were performed in shallow samples. Each experiment was measured on at least n=2 biologically independent samples. Samples showed good agreement to an initial screen for experimental conditions, among similar conditions, and to a broad variation of conditions presented in Supplementary Figure 2 (n=89 experiments pooling N=18 biologically independent samples).

Transcriptomics experiments were conducted using N=3 biologically independent samples. In total, n=9 independent experiments were performed, i.e., a biological triplicate for each experiment. The triplicate, as well as its accompanying proteomics data, showed high reproducibility.

Confocal microscopy micrographs in Extended data Fig. 7 are representative of 10 micrographs taken on n=1 biological sample for each protein. The observation of lack of micron-scale structure was confirmed throughout the other replicates in the FCS and size exclusion bead assays. FCS spectra were collected from N biologically independent samples, measured by n FCS traces across the extract; N/n: Dex70: 2/36, ILF3: 5/36, G3BP2: 1/12, HNRNPA1: 4/26, CIRBP: 4/68, GID8: 4/28, WDR1: 4/23, AP2S1: 3/13, PCF11: 4/23, CCS: 3/33. Confocal micrographs in Fig. 4 and Extended Data Figure 8 are representative for n_1_/n_2_/n_3_/n_4_ micrographs for each bead category of n=1 biological sample, WDR1: 4/4/4/5, GID8: 4/5/5/5, CCS: 4/5/5/4, CIRBP: 4/6/6/5, HNRNPA1: 4/4/4/5, ILF3: 4/4/5/5, MVB12B: 5/4/6/6, QRICH1: 4/2/3/4, VGLL4: 5/4/4/6. Size exclusion assays were performed in n=3 biologically independent replicates showing similar results. Confocal micrographs in Supplementary Figure 4 are representative of 5 micrographs of an in-vitro experiment performed in duplicate. Confocal micrographs in Supplementary Figure 5 are representative for 4 micrographs of n=2 biologically independent samples.

Data distribution was assumed to be normal when performing t-test, but this was not formally tested.

No statistical methods were used to pre-determine sample sizes, but our sample sizes are similar to those reported in previous publications^55,80^.

## Extended Data

**Extended Data Fig. 5:**
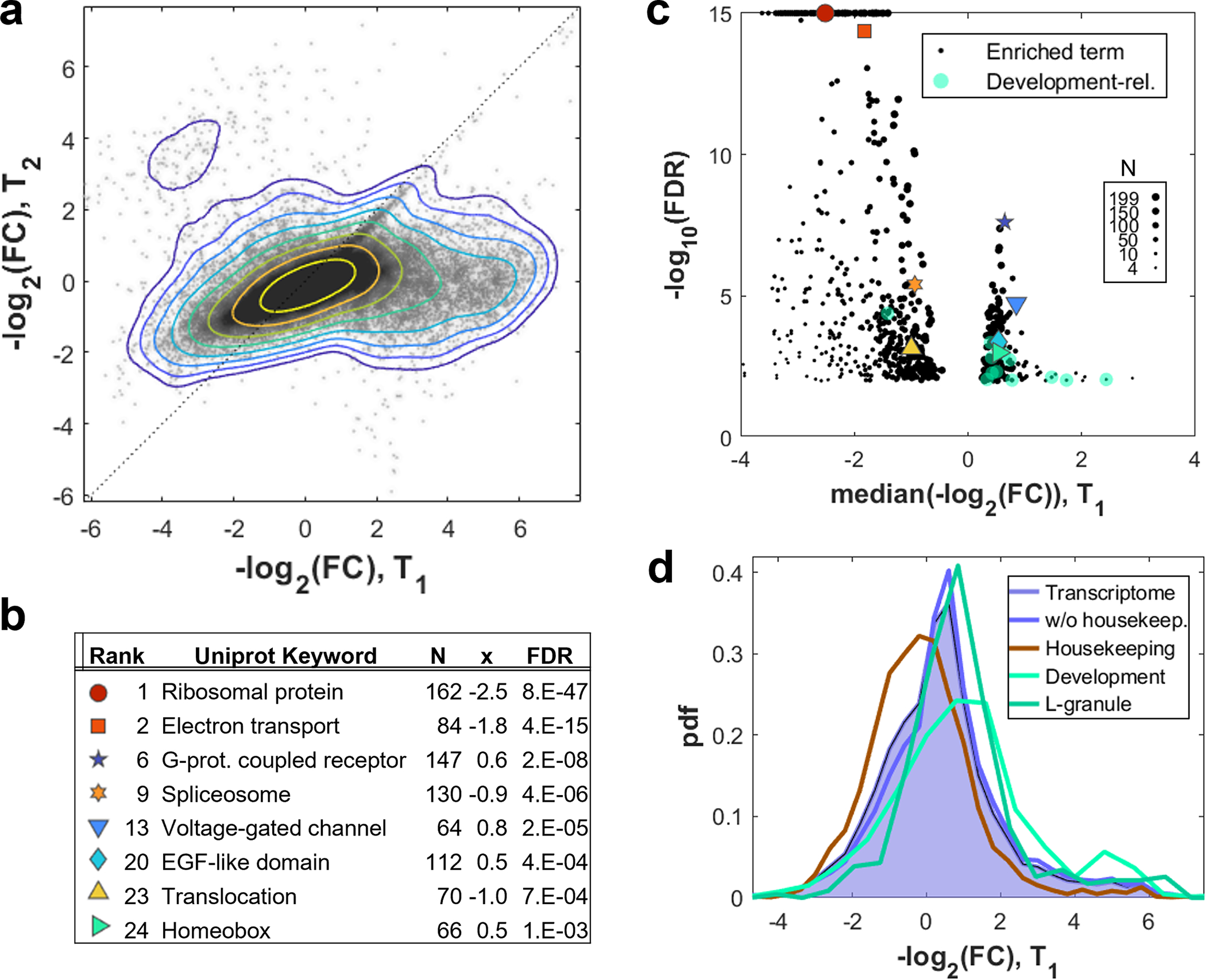
Transcriptomics of filtrated cytoplasm suggests organization for accessibility. **a**, Scatter plot of the transcriptome for a filtration experiment as in Fig. 1c, comparing the foldchanges to the input of an early and a late time T_1_ and T_2_. The majority of RNAs exhibit lightly enhanced flowthrough later on, while about 15% of RNAs are strongly retained at T_1_ but become much more abundant at T_2_. The contour denotes the marker density, each line marking a two-fold increase. FC is the ratio between RNA abundance in transcripts per million reads at T_1_ and T_2_ normalized by the unfiltered condition. N=3 biologically independent replicates. **b, c**, Enrichment analysis of gene database terms (STRING^50^) for the FC at T_1_. The selection of top UniProt Keywords (b) suggests that constitutively translated mRNAs pass the pores easily, while mRNAs for transiently translated are more retained. Highlighting development-related terms in the volcano plot of all enriched terms (c) supports this picture. False discovery rates (FDR) calculated by two sided ks-test^50^. N=3 biologically independent replicates. **d**, Histogram of filtration retention values for the transcriptome. Housekeeping gene transcripts^31^ are shifted towards easier flowthrough (p=3.3e-78), while the cumulative of all development-related terms from (c) have mRNAs in the retained cluster (p=3.9e-29). Similarly enriched are transcripts which are contained in L-bodies^33^ (p=2.4e-11). P-values determined by two-sided t-test. N=3 biologically independent replicates. Source numerical data and transcriptomics data are provided in Source Data and Supplementary Table 3.

**Extended Data Fig. 6:**
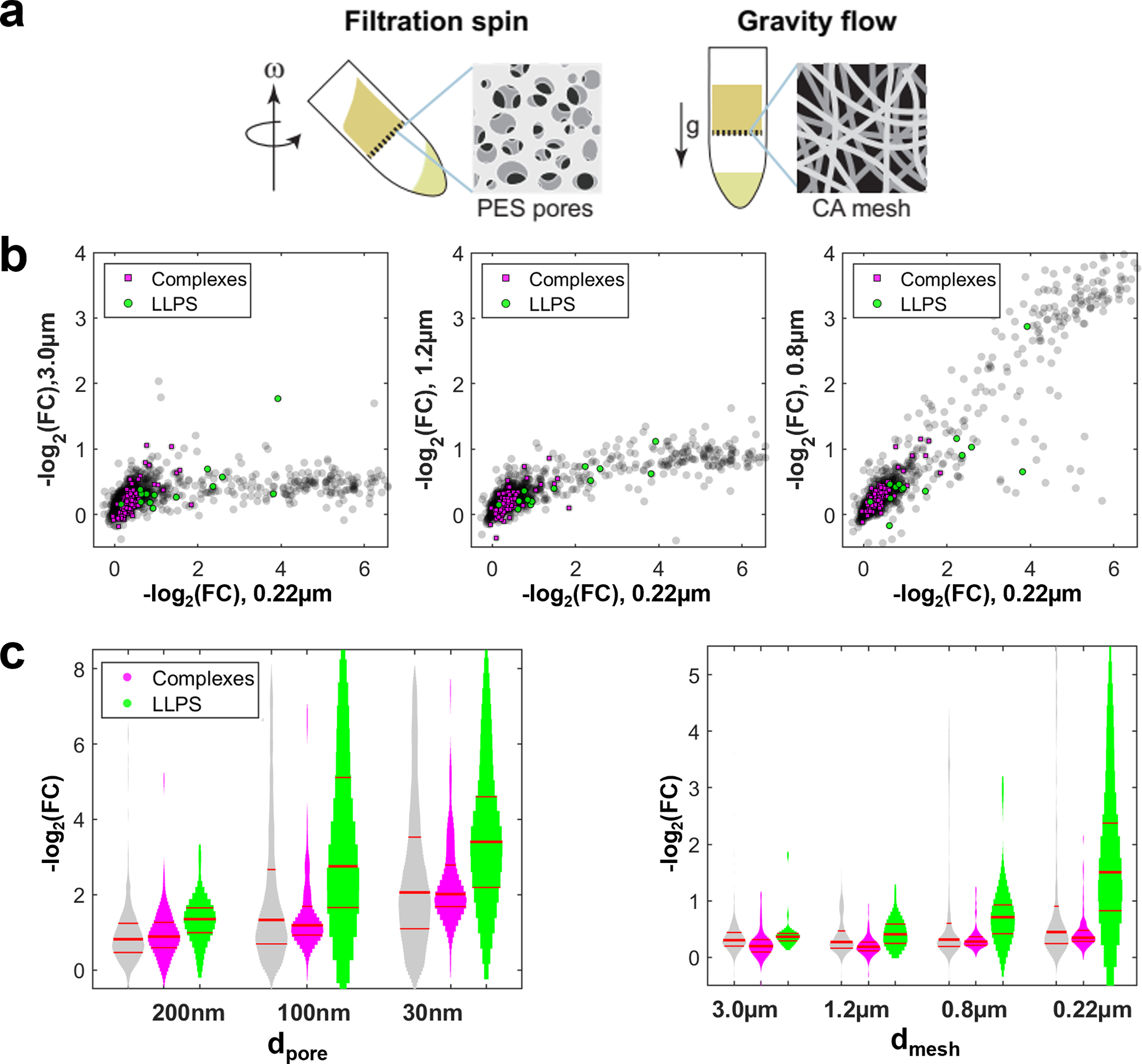
Pore size dependence of filtration. **a,** Schematics of the spin filtration setup, using polyethersulfone (PES) membranes and the alternative setup gravity flow setup, using cellulose acetate (CA)-mesh filters. The large open area of the CA mesh enables flow through at 1g force. **b,** Scatter plots of the fold-changes $FC=c/c_{0}$ in CA gravity flow experiments. Larger meshes (3 μm, 1.2 μm, 0.8 μm; left, mid, right panel on y-axis) can only resolve few structures compared to a 0.22 μm mesh (x-axis). LLPS proteins are shifted to less permeation. N=1 biological sample. **c,** Permeation histograms of the PES filters at the early elution (from main text) (left) and the CA mesh filters (right). The retention increases with smaller pores or meshes, suggesting assemblies on the sub-micrometer length scale. This behavior is pronounced for LLPS proteins. Note that pore and mesh sizes d_pore_ and d_mesh_ are stated as the filter cutoff, i.e., particles larger than this size are confidently retained, and thus most pores or meshes are smaller than this size. However, due to the squeezing behavior of assemblies, we cannot determine a precise size from the cutoff. Red lines in the violin plots denote the 0.25, 0.5, 0.75 quantiles. FCs were normalized separately for each experiment to their 0.95 quantile. N=1 biological sample. Source numerical data and proteomics data are provided in Source Data and Supplementary Table 3.

**Extended Data Fig. 7:**
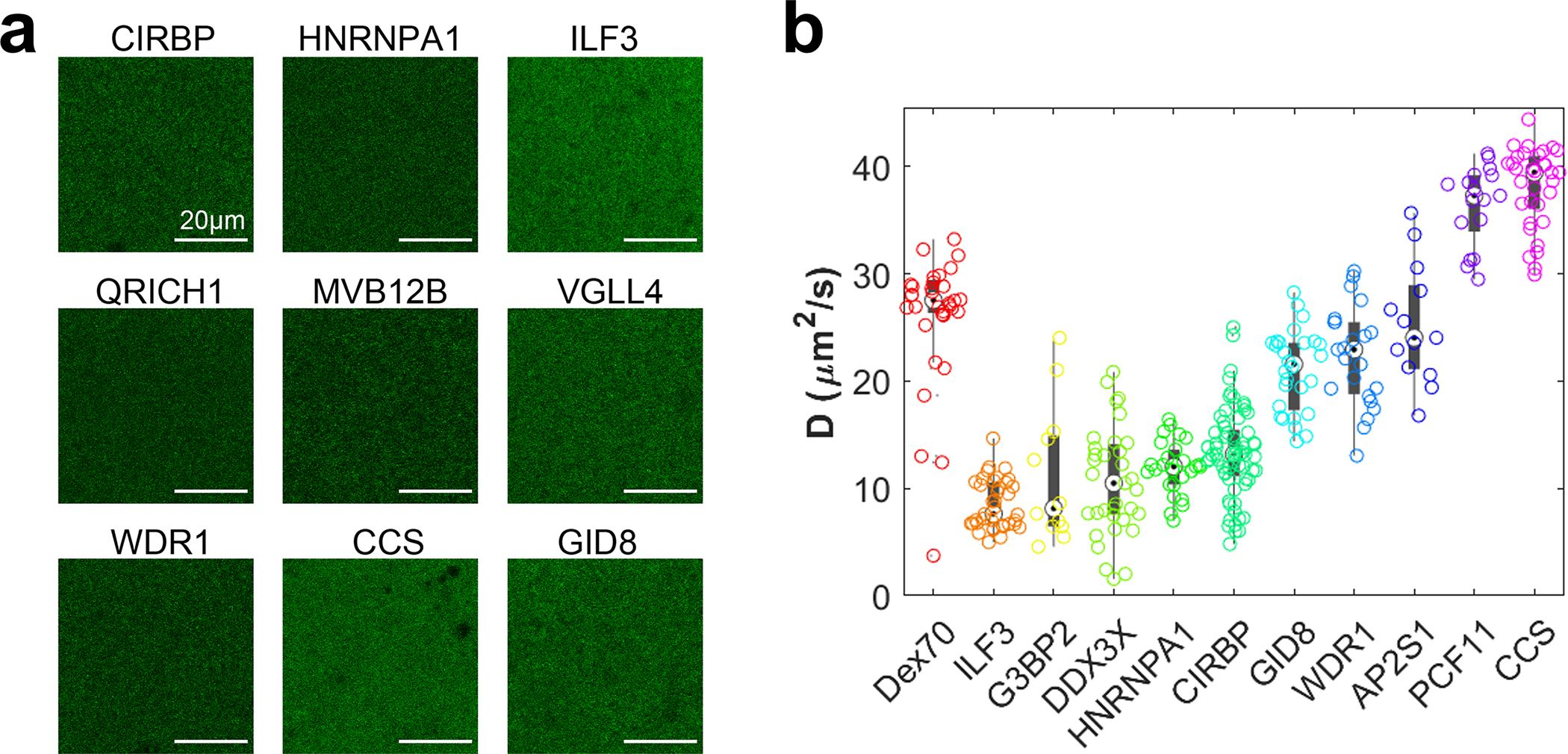
Exemplary proteins organize below the micrometer scale. **a,** Confocal micrographs of GFP-fused proteins expressed from mRNA in the cell extract. Solutions appear relatively homogenous, and we did not detect structures on the micrometer scale. Lookup tables adjusted individually to 0.35% saturated pixels to enhance contrast. **b,** Diffusion constants D of GFP-fused proteins in extract measured by fluorescence correlation spectroscopy. A 70 kDa dextran-rhodamine serves as a reference. Phase separating proteins (ILF3, G3BP2, DDX3X, HNRNPA1, CIRBP) exhibit low diffusion constants, suggesting the presence of assemblies. The measured values would correspond to assemblies with at least tens to hundreds of monomers, based on a rough estimation by the Einstein-Stokes equation and the dextran reference. Boxplots display data distribution with the center as the median, box limits as quartiles, gray dots as outliers, and whiskers to non-outlier extremes. Dex70 (N=2, n=36), ILF3 (N=5, n=36), G3BP2 (N=1, n=12), HNRNPA1 (N=4, n=26), CIRBP (N=4, n=68), GID8 (N=4, n=28), WDR1 (N=4, n=23), AP2S1 (N=3, n=13), PCF11 (N=4, n=23), CCS (N=3, n=33); where N denotes the number of biological samples, and n denotes the number of FCS measurements. Source numerical data are provided in Source Data and Supplementary Table 3.

**Extended Data Fig. 8:**
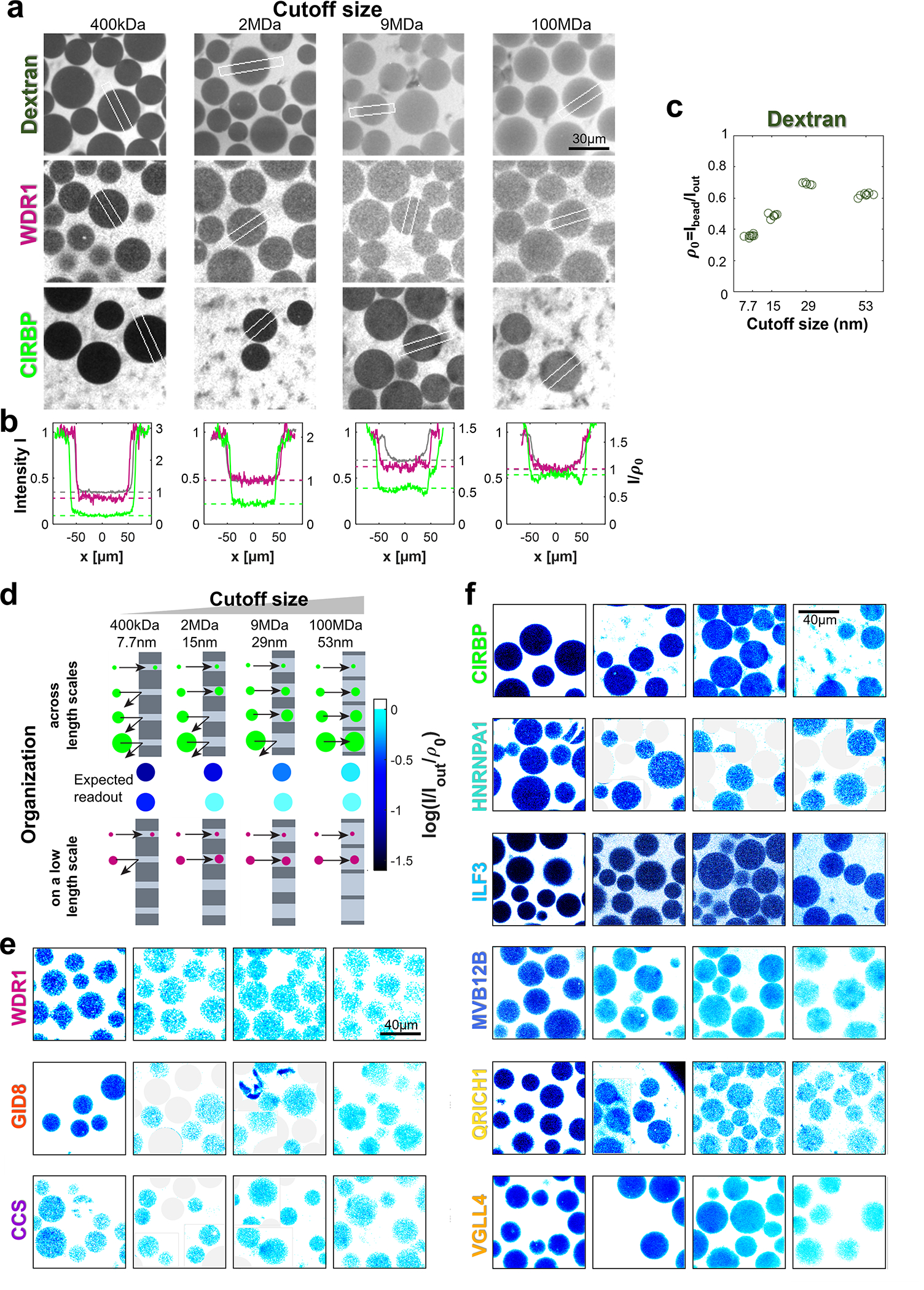
Size exclusion assay. **a,** Confocal micrographs of the assay described in the main text illustrating the measurement. Chromatography beads of different cutoff sizes are placed in extracts with GFP-labeled proteins (WDR1 and CIRBP) and Dextran 70 kDa-rhodamine solution for the calibration measurement. Intensities are normalized to the outside solution. White rectangles indicate regions of the line plots in panel (b). Intensities are normalized to the outside solutions $I_{out}$ for comparability. **b,** Density comparison of the solutions above. Left y-axis displays the normalized, background corrected fluorescence intensity I. Right y-axis displays the intensity normalized to the dextran density $I/\rho_{0}$, as done to correct for accessible volume. **c,** Measurement of dextran intensity ratios that serve as calibration of the assay. The bright, homogenous solutions allow for a precise determination of $\rho_{0}$. **d,** Schematic illustrating the estimate of the size distribution from the exclusion of assemblies from beads with different cutoff sizes. If all assemblies can enter a bead, a fill fraction $f=I/\left({I_{out}\rho }_{0}\right)$ of 1 (cyan) is expected. Exclusion of assemblies means lower f. We observe organization happening either on the 10nm scale (jump of f) or spanning across scales (gradual increase f). **e, f,** Bead assays for the proteins analyzed in Fig. 4d. Color scale as in panel (d). In case of multiplexed assays, beads with different pore size are masked by the gray circles. Insets are provided to show more beads per image. Source numerical data are provided in Source Data and Supplementary Table 3.

**Extended Data Fig. 9:**
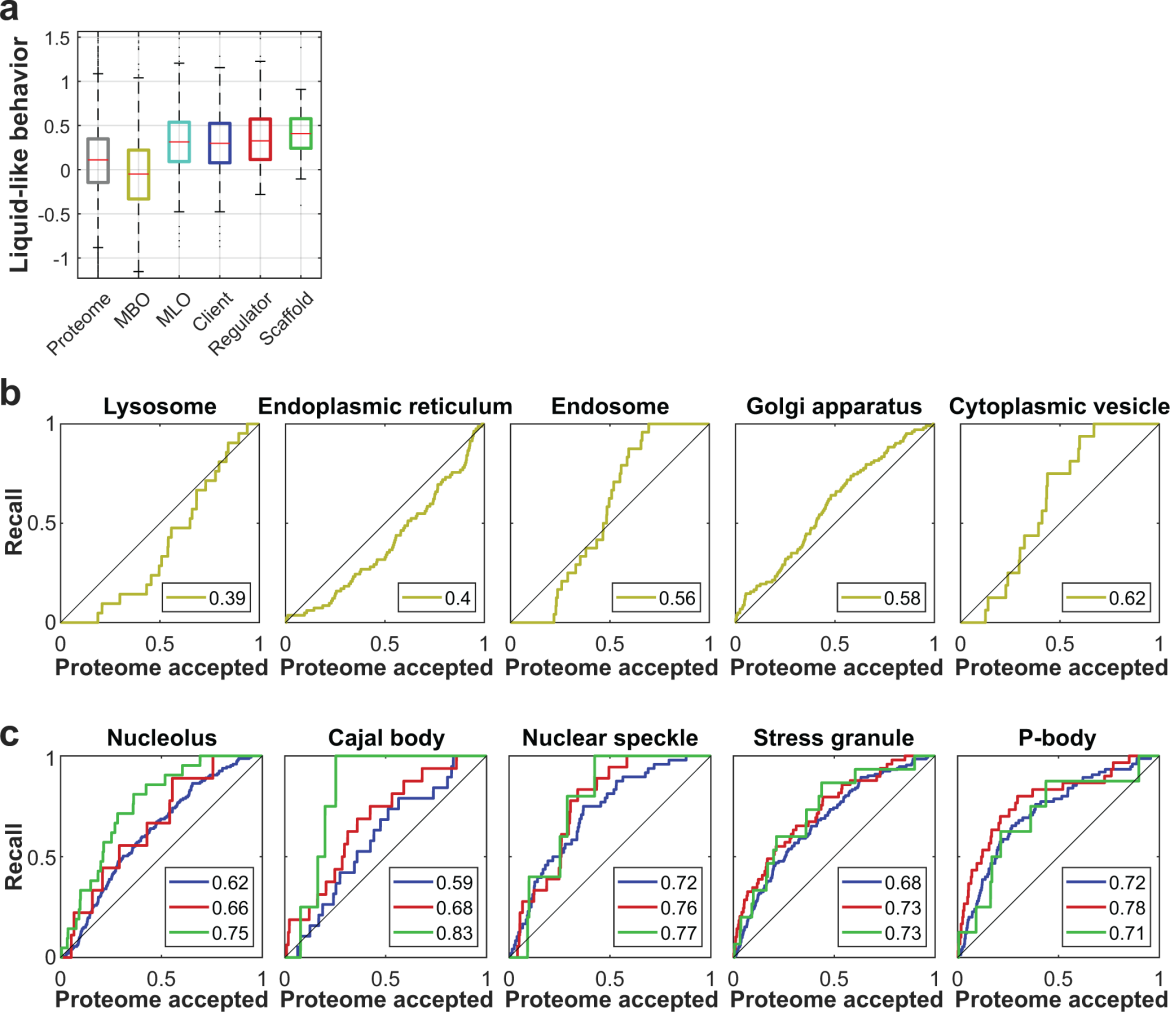
Membrane-bound organelles do not exhibit the typical filtration behavior of BMCs. **a,** Summary of liquid-like behavior, the integrated result of filtration and dilution experiments (n=7 measurements pooled from N=2 biological independent samples). While both distributions for MBOs and BMCs are wide, they are strongly centered on opposite sides of the scale. Scaffold proteins of BMCs are typically more shifted than co-proteins, termed clients and regulators^24^. Boxplots display data distribution with the center as the median, box limits as quartiles, gray dots as outliers, and whiskers to non-outlier extremes. **b**, **c**, ROCs of organelles. Legends display the AUC, color-code as in (a). (c) Most MBOs -with exemption of the Golgi- are depleted from the most liquid-like region of the proteome. (d) BMCs show a good recall characteristic. Typically, co-proteins have lower AUC, as expected from the picture that scaffolds get populated by them depending on the context. Source numerical data and proteomics data are provided in Source Data and Supplementary Table 3.

## Supplementary Material

Source_data_ED_1

Source_data_ED_3

Source_data_ED_2

Source_data_ED_4

Source_data_ED_6

Source_data_ED_7

Source_data_ED_5

Source_data_ED_8

Source_data_ED_9

Source_data_figure_1

Source_data_figure_2

**Figure 3:**
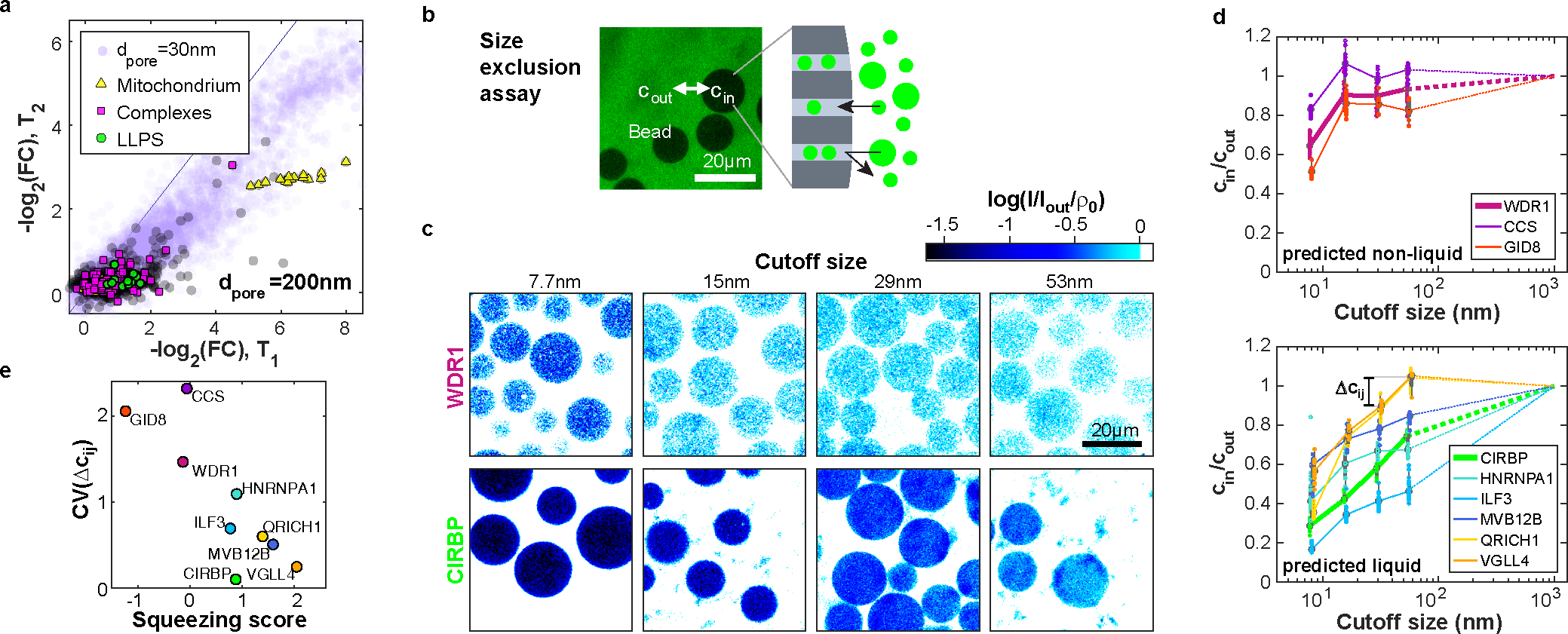
Proteomics and imaging assays indicate that BMCs predominantly organize at the ~100 nm length scale. **a,** The filtration experiment with 200 nm pore size indicates that few proteins besides mitochondrial ones (yellow) are retained (30 nm data for comparison (lilac)). N=1 biological sample. **b,** Microscopy of GFP-labeled proteins with size exclusion beads to assess protein assembly sizes. The concentration inside a bead’s polymer matrix $c_{in}$ compared to outside solution $c_{out}$ provides information about the protein’s assembly size. The example image shows beads with 400 kDa size cutoff in an extract expressing CCS-GFP. N=1 biological sample. **c,** Examples of size exclusion assay for the proteins WDR1-GFP and CIRBP-GFP (examples of proteins in small assemblies and LLPS respectively) with four different bead cutoff sizes. Confocal image intensities I are normalized to their respective outside solution intensity $I_{out}$ and each column is scaled by the accessible volume for monomers $\rho_{0}$ of the used bead. Thus, white bead color stands for $c_{in}\;/c_{out}\approx 1\;$ and darker blue tones report higher exclusions. **d,** Size-exclusion plots. Proteins devoid of squeezing behavior (top panel) show a characteristic jump at short length scale. In contrast, proteins filtrations suggested to be in BMCs (ILF3, MVB12B, QRICH1, VGLL4) including well-established LLPS (CIRBP, HNRNPA1) show organization throughout the meso-scale (bottom panel). Dotted lines extrapolate data to 1 at 1 μm based on lack of visible organization via microscopy. Boxplots denote the median (circle), 0.25/0.75 quantiles (box) and 0.05/0.95 quantiles (whiskers). N=3 biologically independent samples examined over {n_1_,n_2_,n_3_,n_4_} measurements; WDR1: {21,21,21,19}, GID8: {20,30,9,18}, CCS: {20,21,11,23}, CIRBP: {8,7,7,7}, HNRNPA1: {19,9,7,8}, ILF3:{15,11,17,20}, MVB12B: {16,10,20,16}, VGLL4: {15,13,13,4}. **e,** The coefficient of variation (CV) of subsequent steps $\Delta c_{ij}$ of the curves in (d) serves as a metric for the organization patterns. Proteins with a high squeezing score (Fig. 1d) that are predicted to be in liquid-like assemblies exhibit a low CV, suggesting an assembly size distribution spanning the meso-scale. Source numerical and proteomics data are provided in Source Data and Supplementary Table 3.

Source_data_figure_3

**Figure 4:**
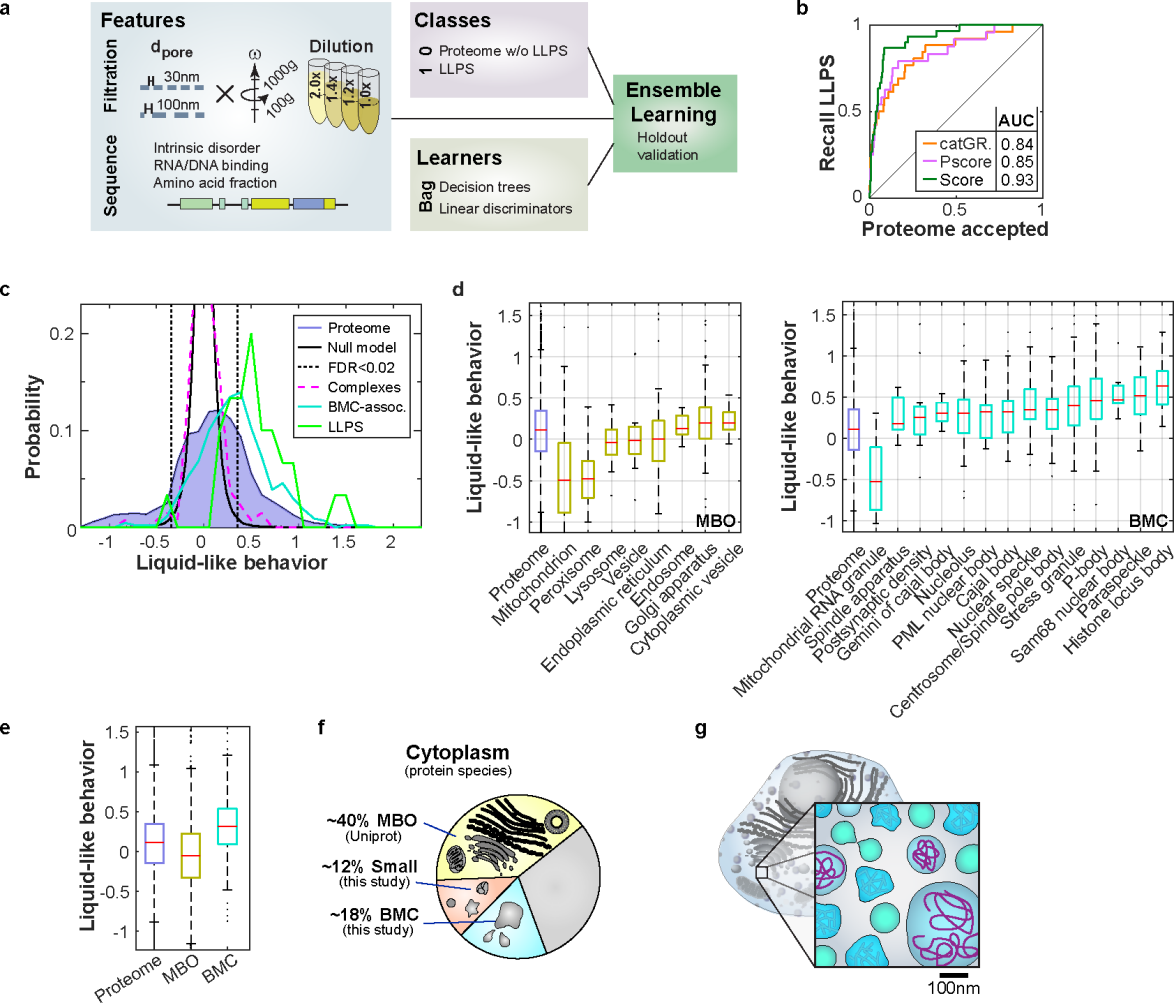
Integrating proteomics experiments enhances LLPS prediction and classifies at least 18% of the cytoplasm as BMC. **a,** Classification ensemble learning to distinguish LLPS proteins from the bulk proteome. We use our deep proteomics data of filtration chromatography (Fig. 1, N=1 biological sample) and diluted filtration chromatography (Fig. 2, N=1 biological sample) and additional sequence annotations as features. Bagging of decision trees and linear discriminators returns the classification score. **b,** ROC displaying the recall of LLPS proteins by the respective scoring. The AUC of our prediction score (dark green) is higher than the one of the established predictors catGRANULE^45^ (orange) and Pscore^46^ (purple) and shows a steeper increase. **c,** We define liquid-like behavior as the accumulative shift in dilution and filtration experiments (n=7 measurements pooled from N=2 biological independent samples), where the null-model (black) is the replicate noise centered around strongly retained assemblies (complexes (magenta) and transmembrane domain proteins). A quarter of the proteome (blue, filled) and 68% of the LLPS references (green) are beyond a 2% false discovery rate (black dotted lines). BMC-associated, supposably not LLPS driving, proteins (teal) are also shifted towards this regime. **d,** Distributions of liquid-like behavior for canonical MBOs (left) and BMCs (right). MBOs’ different liquid-like behaviors agree to the picture that mitochondria are large and stable, and by contrast the Golgi apparatus’ intricate structures partially squeeze through filters. BMCs in general exhibit higher liquid-like behavior, but also show broad distributions. **e,** Accumulatively, BMCs and MBOs exhibit distributions distinct from the proteome. Boxplots (d, e) display data distribution with the center as the median, box limits as quartiles, gray dots as outliers, and whiskers to non-outlier extremes. **f,** Eukaryotic cytoplasmic organization to a large extent is achieved by membrane-bound organelles (~40% of our sample, yellow, UniProt). In our filtration experiments, only around 12% of protein species stay unaffected (salmon). Based on the 2% FDR and correcting for MBOs, 18% of the cytoplasm is organized in BMCs (cyan). We cannot confidently assign any of these three organization modalities for the remaining third of the proteome (gray). **g,** Our results suggest that BMCs frequently have stable cores, contain RNAs, and contribute significantly to cytoplasmic organization at the ~100 nm scale. This indicates that cytoplasm is widely structured at the mesoscale. Source numerical and proteomics data are provided in Source Data and Supplementary Table 3.

Source_data_figure_4

Supplementary_tables

Supplementary information
