# Supplementary material for: Evidence for widespread cytoplasmic structuring into mesoscale condensates
Source: Nat Cell Biol. Author manuscript; Available in PMC 2025 Mar 1. (PMC10981939; doi:10.1038/s41556-024-01363-5)
Supplement: Supplementary information [file NIHMS1974258-supplement-Supplementary_information.pdf]

Supplementary Figures

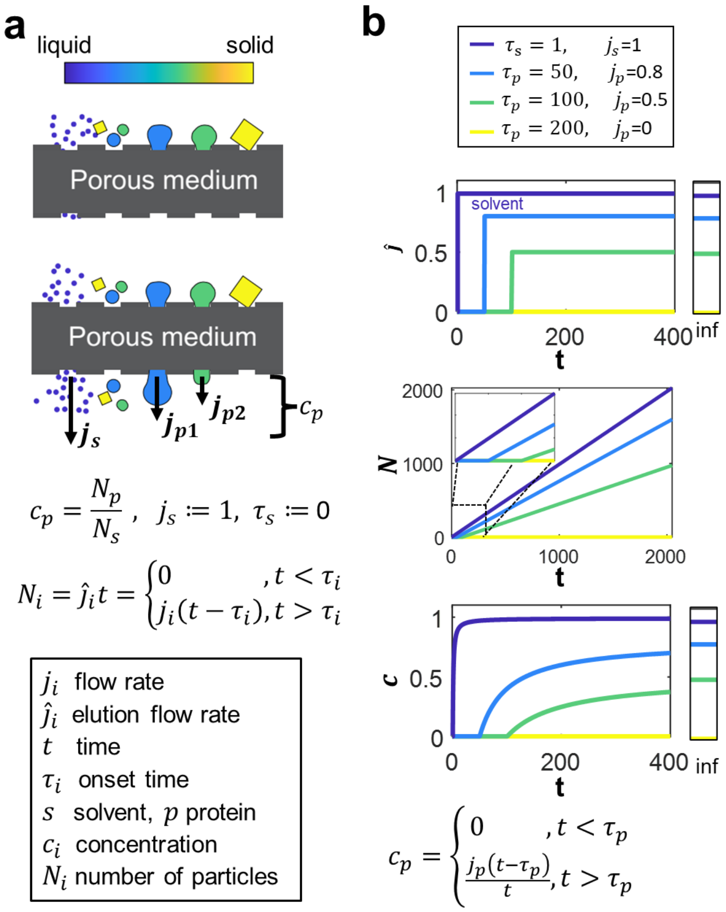

Supplementary Figure 1: Model for the elution measurement. a, Assemblies pass the porous medium with constant flow rates j_i, depending on their size and material properties. In the beginning of the filtration process, this leads to an onset time τ_i for the elution to start, denoted as the elution flow $\hat{j}_i$. Before τ_i the assemblies progress through the medium. For the solvent, τ_s is set to zero, and its flow rate j_s is set to one [count/time-unit]. The measured quantity is the protein concentration in the filtrate $c_p = \frac{N_p}{N_s}$, which is the fraction of protein count per solvent count. These counts are the time integrals of the elution flows.

$$c_p = \begin{cases} 0 & , t < \tau_p \\ \frac{j_p(t-\tau_p)}{t}, & t > \tau_p \end{cases} \qquad (1)$$

b, Simulated flows and concentrations for the example in (a). *Top:* The elution flows are assumed to be step functions at the respective onset time for an assembly. *Mid:* The number of eluted particles N_i is the time integral of the flow. On long time scales all N_i increase linearly. However, due to the onset times these lines do not meet in the origin (inset), *Bottom:* Concentration measurements return the ratio of eluted particles to the solvent $c_p = \frac{N_p}{N_s}$, see Eq. 1 . This results in hyperbolic curves with an offset that go asymptotically towards the elution flow.

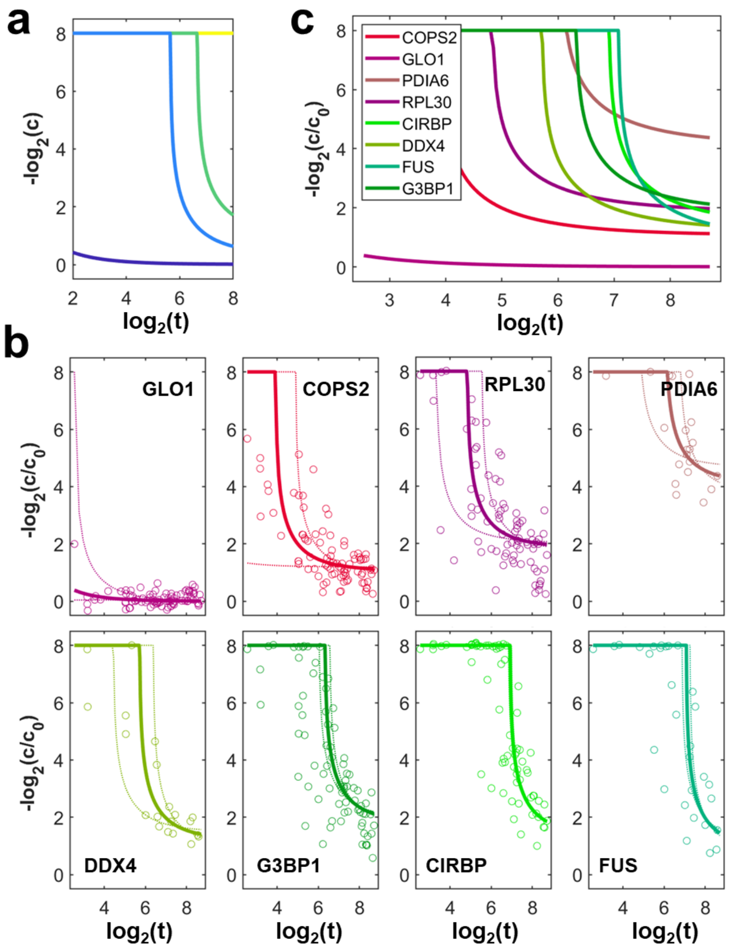

Supplementary Figure 2: Model fit to the data. a, Logarithmic representation of concentration vs. time (b). **b,** Fits of the model to proteomics data of N=89 elution experiments varying spin duration and applied pressure. Proteins exhibit a wide variety of elution behaviors. LLPS proteins (green colors) typically show long onset times and an medium final concentration. A time coordinate t was derived from the total elution mass (see Supplementary Figure 3 b, c). Fits are performed in $c_p t$-space to avoid fitting the steep part of the hyperbolas.

$$c_p t = j_p(t - \tau_p), \ \ t > \tau_p \qquad (2)$$

Fits were performed using MATLAB's 'fit' function using the nonlinear least squares method and a bisquare robust fit. Dotted lines denote the upper and lower fit boundaries.

c, Merged plot of the examples in (b). Both data and fit were capped at 2^{-8}, the assumed detection limit.

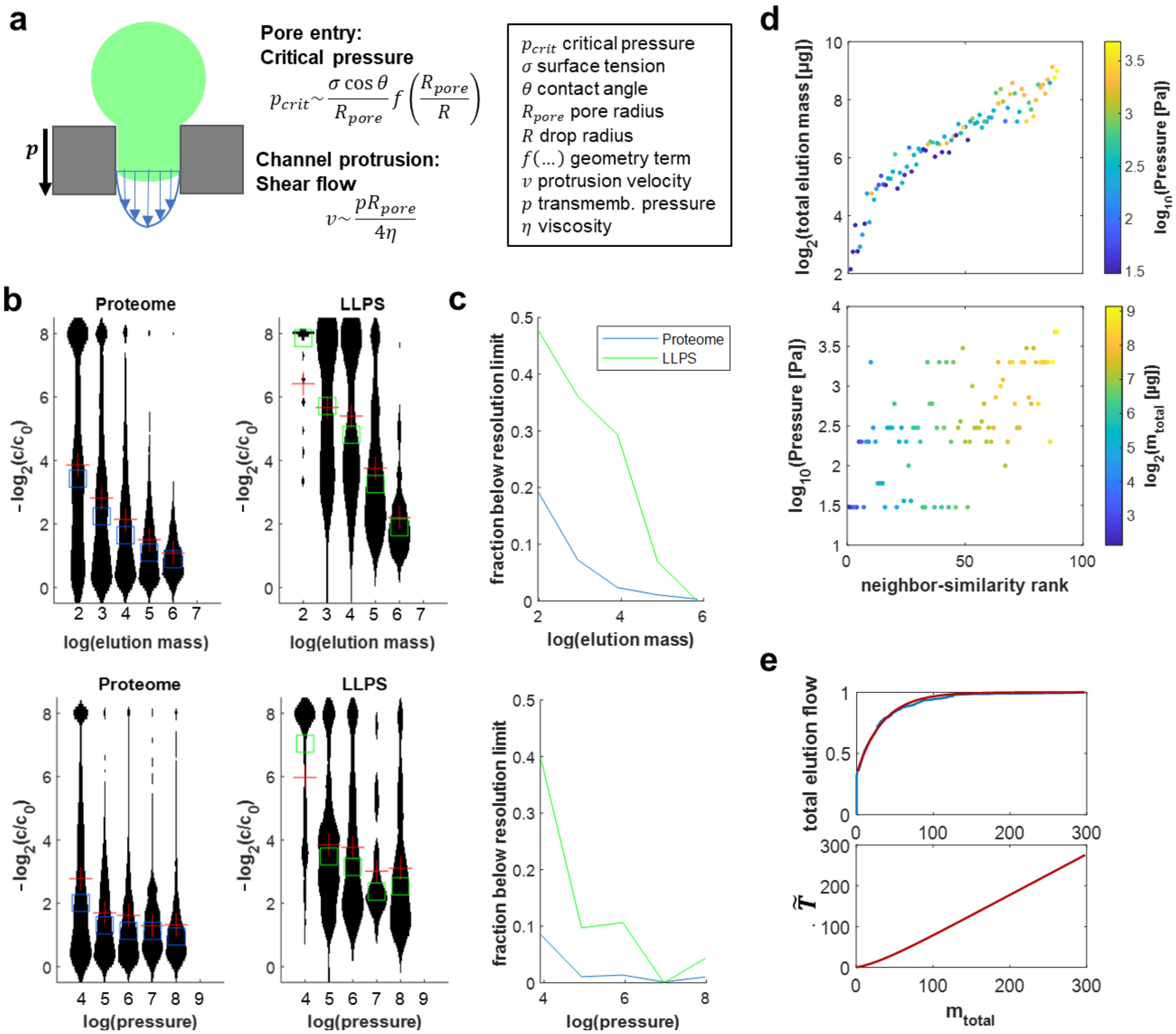

Supplementary Figure 3: Underlying physical processes for elution times of liquid droplets. As a simplified physical model, we consider the pore entry and the protrusion inside the channel as the primary determinants of passage velocity for assemblies larger than pore size. **a,** According to Young-Laplace-based theory, pore entry occurs if the transmembrane pressure exceeds a critical value p_{crit}, which is proportional to surface tension σ. Assuming a Newtonian fluid, the velocity for the protrusion inside the channel v is proportional to the shear rate, and thus to the transmembrane pressure p, the pore size R_{pore}, and the inverse viscosity η^{-1}. Additionally, the entry process creates friction slowing down the protrusion[1]. **b,** Elution histograms grouped by total elution mass and applied pressure. Red crosses and colored squares depict mean and median values. With increasing total elution mass (top), both the whole proteome's and the LLPS proteins' elution rises gradually. This is similar if grouped by applied pressure (bottom). **c,** The fraction of fully retained proteins ($-\log_2(c/c_0) \geq 8$) shows a similar picture as in the histograms (b): In the experimentally accessible pressure range, there is no general critical pressure behavior for LLPS proteins, however the data suggests critical pressures may exist and are low. **d,** Comparison of filtration results with respect to applied pressure and total eluted mass m_{total}. The two plots display the same quantities on alternate

axis. Rank ordering of filtration experiments (R_{pore} =30nm, N=89) by their pairwise similarity of elution spectra ("neighbor similarity rank") highly correlates with total elution mass collected in the filtrates, but only poorly with applied pressure. If we consider pore passage to be set by a viscosity dependent shear flow and a surface tension dependent critical pressure (see (a)), this data suggests the shear flow term is dominant. (Note that the highest masses can only be obtained at high pressures in reasonable acquisition time, increasing the correlation.) **e,** Definition of an elution time coordinate t The total elution flow at each elution mass m_{total} (blue), is given by $J = \sum_i c_i j_i$, where c_i are the protein concentrations from literature. As m_{total} is the integrated total flow over time $m_{total} = \int_0^t J dt$, a time coordinate t can be defined for the flow model to map m_{total} to a linear time axis (red). In the experiments, long-time acquisitions are taken at higher pressures, effectively speeding up the time.

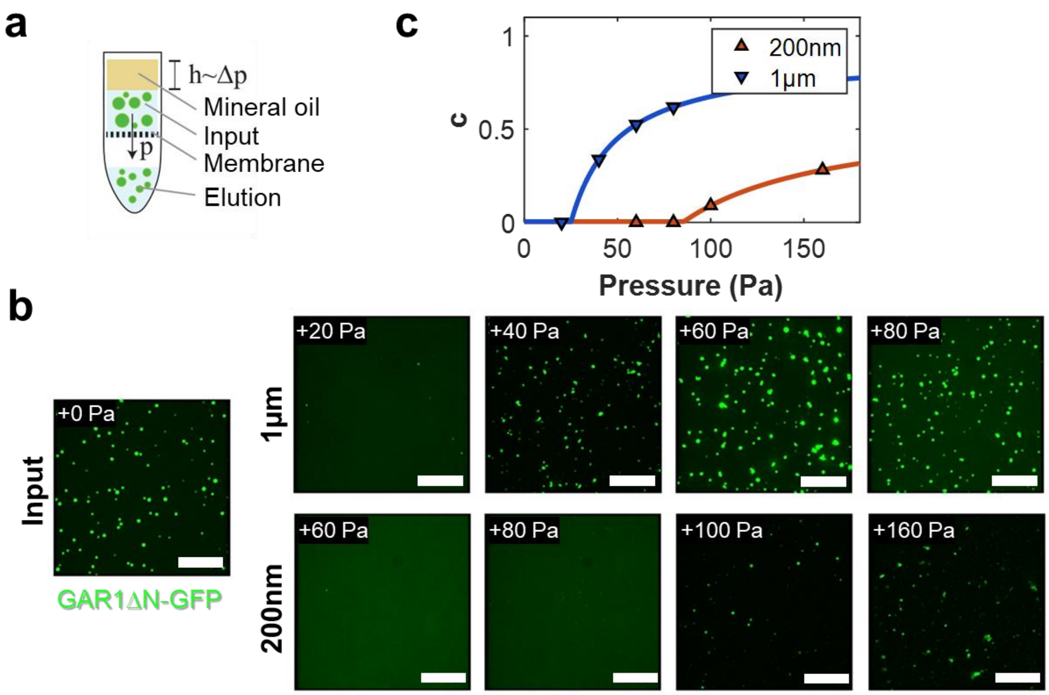

Supplementary Figure 4: In vitro setup testing filtration of liquid droplets. a, In-tube filtration setup. An in vitro solution of phase-separated GAR1ΔN-GFP droplets flows through a porous membrane. Gravity drives the flow, and the applied pressure is varied by the height of a layer of mineral oil. Filtrates are collected at fixed elution time. **b,** Fluorescence micrographs corresponding to the experiment described in (a), using pore sizes of 1 μm (top) and 200 nm (bottom) to filter the input solution (left). Bars are 20μm. **c,** Schematic summary of the observed elution. With increasing pressure, more droplets from the input pass the filters. Qualitatively, this corresponds to increasing elution over time at fixed pressure if critical pressure behavior is negligible. With the 200 nm pore size, as expected, the onset happens at higher pressures and only smaller droplets pass.

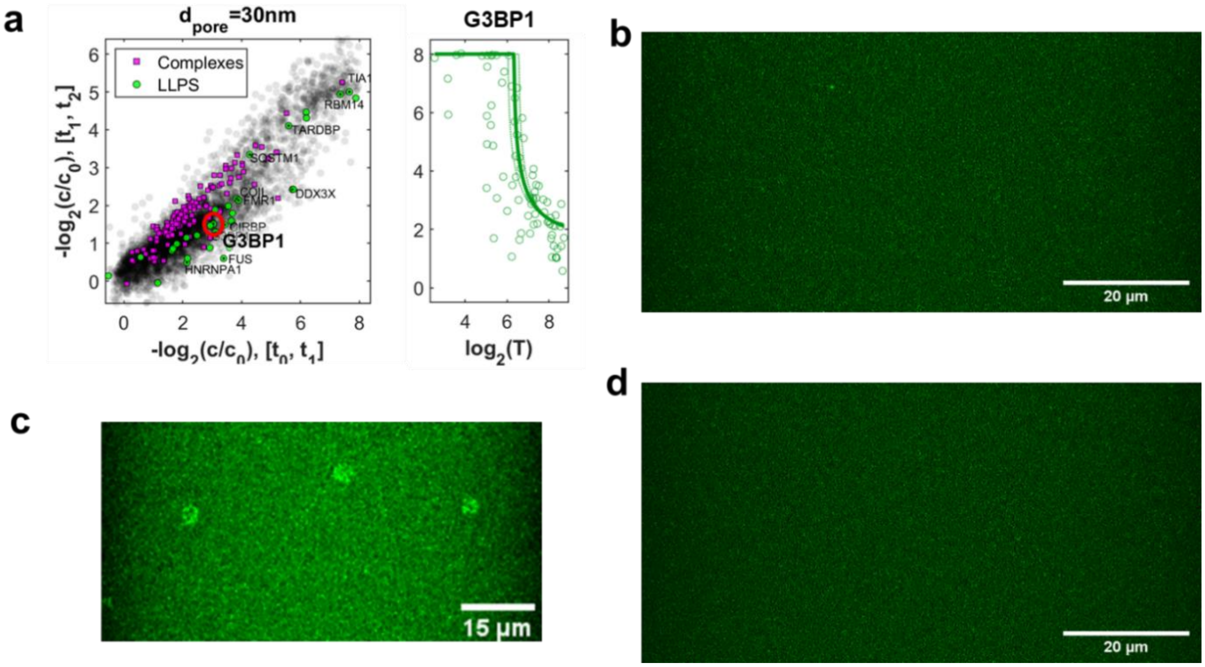

Supplementary Figure 5: **Filtration of artificially induced assemblies. a,** G3BP1, the scaffold of stress granules, behaves like a typical liquid assembly in our mass-spectrometry filtration assays. Dotted lines denote the upper and lower fit boundaries. **b,** Confocal microscopy data of G3BP1-GFP expressed from mRNA in cell extract shows no organization on the micron scale. **c,** Upon addition of Sodium Arsenide, assemblies form and merge (laser intensity and gain adjusted for improved visibility). **d,** Filtration of the solution from (c) after assembly induction (presumably stress granules) with arsenide addition through 100 nm pores yields a solution with no visible structure. The image has similar intensity statistics as the input.
